# Revisiting the taxonomy of the Rattini tribe: a phylogeny-based delimitation of species boundaries

**DOI:** 10.1186/1471-2148-10-184

**Published:** 2010-06-18

**Authors:** Marie Pagès, Yannick Chaval, Vincent Herbreteau, Surachit Waengsothorn, Jean-François Cosson, Jean-Pierre Hugot, Serge Morand, Johan Michaux

**Affiliations:** 1 INRA, UMR CBGP (INRA/IRD/Cirad/Montpellier SupAgro), Campus International de Baillarguet, CS 30016, 34988 Montferrier-sur-Lez cedex, France; 2 Cemagref, Territories, Environment, Remote Sensing and Spatial Information Joint Research Unit (UMR TETIS), Maison de la Télédétection, 500 rue J-F Breton, 34093 Montpellier Cedex 5, France; 3 Institut de Recherche pour le Développement (IRD), Research Unit UR178, Center for Vectors and Vector-borne Diseases (CVVD), Faculty of Sciences, Mahidol University, Bangkok 10400, Thailand; 4 Thailand Institute of Scientific and Technological Research, Bangkok, Thailand; 5 Muséum National d'Histoire Naturelle, Origine, Structure et Evolution de la Biodiversité, Paris, France; 6 Institut des Sciences de l'Evolution, CNRS-UM2, Université Montpellier 2, 34095 Montpellier, France; 7 UR AGIRs, CIRAD, Campus International de Baillarguet 34398 Montpellier, France; 8 Laboratoire de génétique des microorganismes, Institut de Botanique, Université de Liège, 4000 Liège (Sart Tilman), Belgique

## Abstract

**Background:**

Rodents are recognized as hosts for at least 60 zoonotic diseases and may represent a serious threat for human health. In the context of global environmental changes and increasing mobility of humans and animals, contacts between pathogens and potential animal hosts and vectors are modified, amplifying the risk of disease emergence. An accurate identification of each rodent at a specific level is needed in order to understand their implications in the transmission of diseases. Among the Muridae, the Rattini tribe encompasses 167 species inhabiting South East Asia, a hotspot of both biodiversity and emerging and re-emerging diseases. The region faces growing economical development that affects habitats, biodiversity and health. Rat species have been demonstrated as significant hosts of pathogens but are still difficult to recognize at a specific level using morphological criteria. DNA-barcoding methods appear as accurate tools for rat species identification but their use is hampered by the need of reliable identification of reference specimens. In this study, we explore and highlight the limits of the current taxonomy of the Rattini tribe.

**Results:**

We used the DNA sequence information itself as the primary information source to establish group membership and estimate putative species boundaries. We sequenced two mitochondrial and one nuclear genes from 122 rat samples to perform phylogenetic reconstructions. The method of Pons and colleagues (2006) that determines, with no prior expectations, the locations of ancestral nodes defining putative species was then applied to our dataset. To give an appropriate name to each cluster recognized as a putative species, we reviewed information from the literature and obtained sequences from a museum holotype specimen following the ancient DNA criteria.

**Conclusions:**

Using a recently developed methodology, this study succeeds in refining the taxonomy of one of the most difficult groups of mammals. Most of the species expected within the area were retrieved but new putative species limits were also indicated, in particular within *Berylmys *and *Rattus *genera, where future taxonomic studies should be directed. Our study lays the foundations to better investigate rodent-born diseases in South East Asia and illustrates the relevance of evolutionary studies for health and medical sciences.

## Background

Among mammals, rodents are recognized as major hosts and vectors of parasites and pathogens, some of them causing important zoonoses and representing a serious threat for human health [1-5]. Most epidemiological studies have focused on the most common rodents with emphasis on commensal species such as the laboratory rat, *Rattus norvegicus*. A common assumption is that the rodent species responsible for disease transmission are those living close to humans, but since wild species distant from human settlements have been proven to play a key role in maintaining, spreading and transmitting pathogens and parasites (e.g. [4]), this point of view is being questionned. Specific diversity within the host community has also been shown to play an important function in the maintenance of a disease and in the probability of its transmission to humans [6,7]. Consequently, researchers are now focusing not on a single particular host species but on the whole host community and are endeavouring to understand the role of each rodent species in the context of the entire host-pathogen community.

Today this knowledge is more urgent than ever since biodiversity in many areas is being altered rapidly by the ongoing global change. Because of anthropogenic disturbances, the host-pathogen interactions are being dramatically modified leading to new and unexpected disease risks and the emergence and/or re-emergence of infectious diseases [6-10]. To be able to predict and to anticipate some of these risks, one should be able in the case of rodent host communities, to identify first and foremost each rodent at a specific level, a real challenge when considering that rodents represent 40% of mammalian species [11] including many cryptic species, and that new genera and species are yearly described (*e.g. Laonastes aenigmamus*, [12]; *Saxatilomys paulinae*, [13]; *Mayermys germani*, [14]; *Tonkinomys daovantieni*, [15]).

Among Muridae rodents, the Rattini tribe encompasses 35 genera corresponding to 167 rat species [16] following the tribal arrangement of the Murinae proposed by Lecompte *et al. *[17]. Nearly all representatives of this tribe inhabit South East Asia, a major hotspot of biodiversity [18] faced with a runaway economic growth damaging habitats, biodiversity and health but also a hotspot of emerging and re-emerging diseases [19,20]. If the partition of the tribe among five divisions (*i.e. Crunomys, Dacnomys, Maxomys, Micromys *and *Rattus *divisions) [16,17] is widely accepted, its taxonomy remains however largely untested phylogenetically and its delimitations are not yet secured. *Chiropodomys*, *Vandeleuria, Hapalomys, Haeromys *and *Vernaya *genera were included in the *Micromys *division by Musser and Carleton [16]. As the Eurasian harvest mouse, *Micromys *was proven to belong to the Rattini tribe ([17,21]), the whole *Micromys *division should belong to the Rattini tribe if Musser and Carleton's assumption is right. However, some of these genera (*i.e. Chiropodomys *and *Vandeleuria*) were recently shown to be unaffiliated to *Micromys *according to molecular evidences [21], while putative representatives of the Rattini tribe (*i.e. Tonkinomys daovantieni, Saxatilomys paulinae, Srilankamys sp., Hapalomys sp., Haeromys sp., Vernaya sp.*) have not been investigated using molecular data and are currently considered as Murinae *incertae sedis *[17]. Numerous rat species have been demonstrated or postulated as major hosts of pathogens (*e.g. *Hantaviruses described from bandicoot rat, *Bandicota indica *in Thailand, [22,23]; *Bandicota indica*, *B. savilei*, *Berylmys berdmorei*, *Niviventer sp.*, and *Rattus sp*. serologically tested positive for *Rickettsia tsutsugamushi*, the agent responsible for scrub typhus [24]; etc.). Although easily identified at a generic level by an expert, Asian rats are often difficult to discriminate at a specific level using morphological or cytological criteria. The wide range of intra-specific morphological variation makes morphological criteria unsuitable for accurate rat species identification and has led to an over-description of species and to a confusing taxonomy, hampered by an overabundance of synonyms. It is particularly true concerning the *Rattus *genus (*e.g. *41 synonyms for *R. norvegicus*, 83 for *R. rattus*, etc. [16] and see also [25]) that consists of a heterogeneous accumulation of species and of several monophyletic clusters that may or may not prove to be grouped in a single genus [16]. This polyphyletic pattern is highlighted by the six species groups proposed by Musser and Carleton [16] (*i.e. *the *Rattus rattus*, *Rattus exulans*, *Rattus norvegicus*, *Rattus fuscipes*, *Rattus leucopus *and *Rattus xanthurus *species groups) and a seventh assemblage containing unaffiliated species (*i.e. *the *Rattus *species group unresolved) for which phylogenetic affinities are uncertain; some representatives will eventually be removed from the genus. Even karyotypic criteria, which previously claimed to be species diagnostic tools, were recently revealed to be unsuitable to discriminate between Asian rat species [26]. DNA-based methods, however, appear to be promising tools for easy and accurate rat species-specific identifications [26].

Robins *et al. *[25] were the first to attempt to identify *Rattus *species using mitochondrial DNA sequences mostly obtained from museum tissue samples. Nevertheless, their conclusions based on DNA-barcoding and tree based methods were limited because these methods need reliably identified specimens as reference. Specimens and tissues offered by museums to scientists are collected by many different people and it seems likely, given the extent of some misidentifications, that rat species identification is not an easy task even for mammal specialists. Moreover, the taxonomy of the tribe Rattini is complex and changing and often different to that in use when samples were first described and listed in museums [25].

Level of variation in cytochrome *b *sequences was also proposed as a reference point in making decisions concerning species-level distinctions [27]. Based on the analysis of 4 genera of rodents, Bradley and Baker [27] suggested that genetic distance values lesser than 2% were indicative of intraspecific variation and values higher than 11% of species recognition. But how to conclude between 2 and 11% ? The DNA-based species delimitation approach proposed by Pons *et al. *[28] relies on DNA sequence information itself as the primary information source for establishing group membership and defining putative species and does not require defining entities as priors. This method was shown to be useful for identifying meaningful entities among groups whose current taxonomy is incomplete (*e.g*. tiger beetles of the genus *Rivacindela*, [28]) or uncertain (*e.g*. aphids of the genus *Brachycaudus*) and has already been successfully applied when species are difficult to conceptualize (*e.g. *bacteria [29] or for asexual animals, [30,31]). Using a likelihood framework, this new procedure detects the point of transition in the rate of lineage branching of a tree from interspecific long branches to intraspecific short burgeoning branching and identifies clusters of specimens corresponding to putative species.

In our study, we used molecular data to test the limits of the current taxonomy of the Rattini tribe. We aimed at identifying where species boundaries are unclear and where further investigations need to be carried out to provide a more rigorous systematic framework for epidemiological surveys. As molecular data are useful to detect and distinguish morphologically similar species, this study investigated the existence of putative cryptic species among the Rattini tribe (*i.e*. two or more species that are classified as a single nominal species because they are at least superficially morphologically indistinguishable [32]). To these aims, we first sequenced two mitochondrial and one nuclear genes from rat specimens coming from Southeast Asia (Thailand, Cambodia and Lao People's Democratic Republic) to perform phylogenetic reconstructions. Then, as morphological characters are often misleading, we applied the method developed by Pons *et al. *[28] that determines, with no prior expectations, the locations of ancestral nodes to define putative species. Finally, we endeavoured to give a name to each cluster recognized as a putative species using information from the literature and also sequences obtained from a museum holotype specimen following all the ancient DNA guidelines.

## Methods

### 1. Sampling

**Figure 1:**
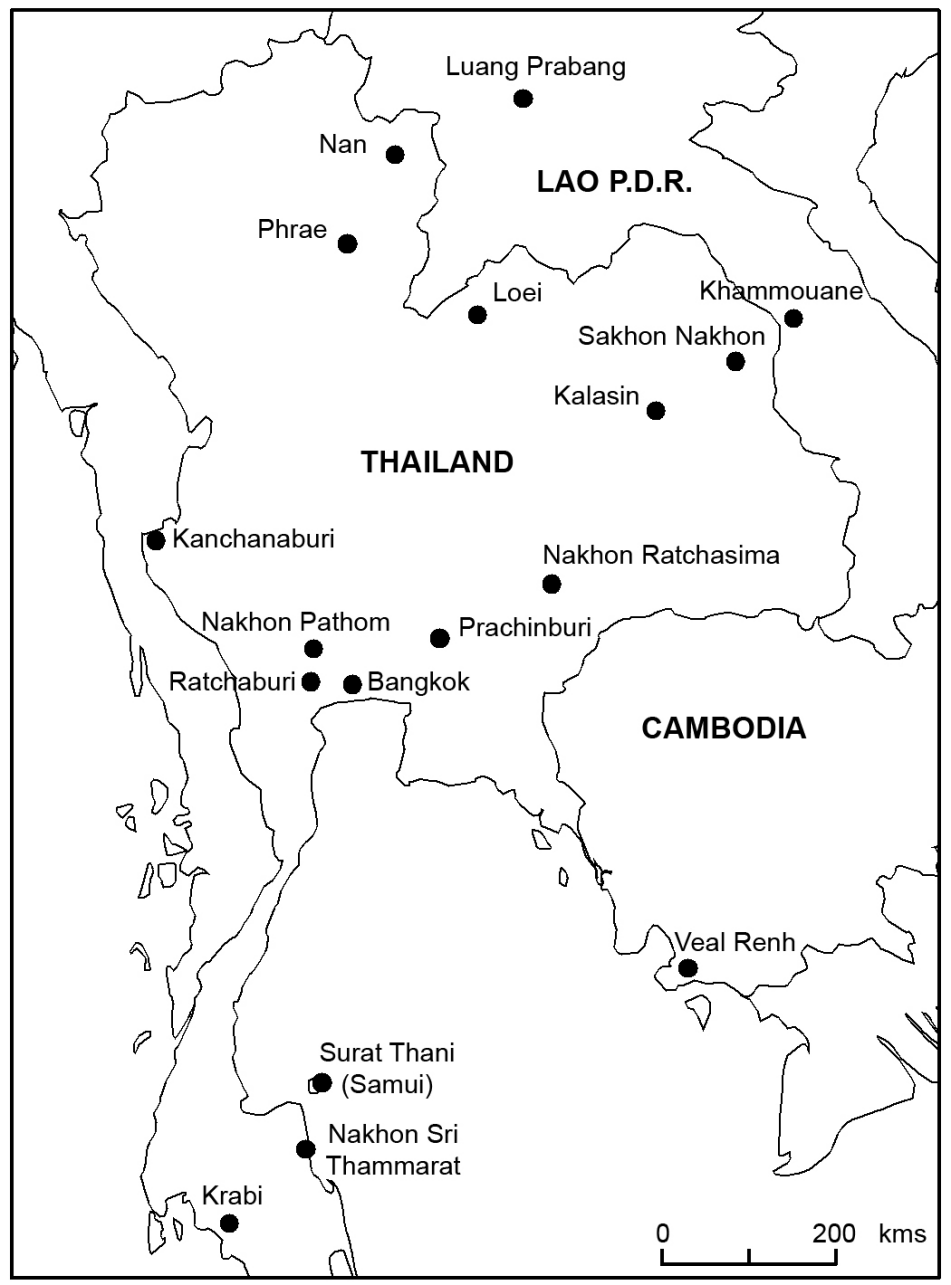
**Sample locations of the Rattini specimens caught in the field and included in this study**. See Table 1 for more sample information.

116 specimens of Rattini were selected among the 3,000 trapped by our team in the fields mostly in Thailand and punctually in Cambodia and in Lao PDR. Specimens selected were chosen in order to maximise the number of species and geographic locations analysed. Field specimen identifications and locality information are listed in Table 1 and indicated in Figure 1. Field identifications were made based on morphological criteria according to [11,33-35]. Based on morphological and cytological evidences, no specimen was identified by us as a representative of the cosmopolitan *Rattus rattus *species. Considering their preponderant place in epidemiological surveys, 4 worldwide black rat specimens (identified in [36]) were added to the sample set. To provide an appropriate outgroup, we included specimens of the Eurasian harvest mouse, *Micromys *belonging to the Rattini tribe and previously recognized as the sister lineage to the *Rattus *group sensu lato of Verneau *et al.*, [37,38,17,21]. In total, our taxa sampling consisted of 122 rats.

**Table 1:** Samples used in this study.

*Sample information*					*GenBank Accession Number*		
Laboratory sample number	Field Identification	Locality	Voucher localisation	Phylogenetic species	Cyt b	COI	IRBP
MDZ10Mada	*Rattus rattus*	Madagascar		R1	HM217368	HM217495	HM217603
ratcosT820	*Rattus rattus*	India		R1	HM217367	HM217498	HM217606
ratcosR12	*Rattus rattus*	Oman		R1	HM217366	HM217496	HM217604
ratcosTE4264	*Rattus rattus*	Tanzania		R1	HM217365	HM217497	HM217605

R4003	*Rattus tanezumi*	Kalasin (Thailand)	MahaU	R2	HM217436	HM217563	HM217673
**R2953**	***Rattus tanezumi***	**Kanchanaburi (Thailand)**		R7	HM217396	HM217525	HM217634
R2996	*Rattus tanezumi*	Kanchanaburi (Thailand)		R2	HM217398	HM217529	HM217636
R3122	*Rattus tanezumi*	Kanchanaburi (Thailand)		R2	HM217407	HM217537	HM217645
R3214	*Rattus tanezumi*	Kanchanaburi (Thailand)		R2	HM217410	HM217540	HM217648
R3573	*Rattus tanezumi*	Nakhon Pathom (Thailand)	KU	R2	HM217430	HM217558	HM217667
R4016	*Rattus tanezumi*	Phrae (Thailand)	CBGP	R2	HM217438	HM217565	HM217675
R4424	*Rattus tanezumi*	Phrae (Thailand)	MahaU	R2	HM217456	HM217582	HM217693
R4436	*Rattus tanezumi*	Phrae (Thailand)	MahaU	R2	HM217457	HM217583	HM217694
R5294	*Rattus tanezumi*	Nan (Thailand)	MahaU	R2	HM217466	HM217592	HM217704
R5296	*Rattus tanezumi*	Nan (Thailand)	CBGP	R2	HM217467	HM217593	HM217705
L0100	*Rattus tanezumi*	Luang Prabang (LPDR)	MahaU	R2	HM217475	HM217489	HM217712
L0194	*Rattus tanezumi*	Luang Prabang (LPDR)	MahaU	R2	HM217480	HM217494	HM217717
**R3029**	***Rattus tanezumi***	**Bangkok (Thailand)**		**R3**	HM217399	HM217530	HM217637
**R1843**	***Rattus tanezumi***	**Krabi (Thailand)**		**R3**	HM217393	HM217524	HM217631
**R1147**	***Rattus tanezumi***	**Nakhon Ratchasima (Thailand)**		**R3**	HM217384	HM217515	HM217622
**R1016**	***Rattus tanezumi***	**Nakhon Ratchasima (Thailand)**		**R3**	HM217382	HM217513	HM217620
**R1818**	***Rattus tanezumi***	**Prachinburi (Thailand)**		**R3**	HM217389	HM217520	HM217627
**R2794**	***Rattus tanezumi***	**Ratchaburi (Thailand)**		**R3**	HM217394	HM217526	HM217632
**R0169**	***Rattus tanezumi***	**Ratchaburi (Thailand)**		**R3**	HM217372	HM217503	HM217610
**CB0028**	***Rattus tanezumi***	**Veal Renh (Cambodia)**	MahaU	**R3**	HM217363	HM217485	HM217601
**R1833**	***Rattus tanezumi***	**Nakhon Sri Thammarat (Thailand)**		R5	HM217391	HM217522	HM217629

R4402	*Rattus losea*	Loei (Thailand)	MahaU	R4	HM217454	HM217581	HM217691
R3484	*Rattus losea*	Loei (Thailand)		R4	HM217421	HM217550	HM217659
R4230	*Rattus losea*	Loei (Thailand)	CBGP	R4	HM217446	HM217573	HM217683
R1015	*Rattus losea*	Nakhon Ratchasima (Thailand)		R4	HM217381	HM217512	HM217619
R4203	*Rattus losea*	Phrae (Thailand)	CBGP	R4	HM217443	HM217570	HM217680
R3510	*Rattus losea*	Phrae (Thailand)		R4	HM217423	HM217552	HM217661
R0237	*Rattus losea*	Ratchaburi (Thailand)		R4	HM217374	HM217505	HM217612
R0238	*Rattus losea*	Ratchaburi (Thailand)		R4	HM217375	HM217506	HM217613

R1805	*Rattus exulans*	Bangkok (Thailand)		R8	HM217388	HM217519	HM217626
R4004	*Rattus exulans*	Kalasin (Thailand)	MahaU	R8	HM217437	HM217564	HM217674
R3224	*Rattus exulans*	Kanchanaburi (Thailand)		R8	HM217411	HM217541	HM217649
R4103	*Rattus exulans*	Loei (Thailand)	MahaU	R8	HM217440	HM217567	HM217677
R1055	*Rattus exulans*	Nakhon Ratchasima (Thailand)		R8	HM217383	HM217514	HM217621
R1836	*Rattus exulans*	Nakhon Sri Thammarat (Thailand)		R8	HM217392	HM217523	HM217630
R4140	*Rattus exulans*	Phrae (Thailand)	MahaU	R8	HM217441	HM217568	HM217678
R0284	*Rattus exulans*	Ratchaburi (Thailand)		R8	HM217377	HM217508	HM217615
R2795	*Rattus exulans*	Ratchaburi (Thailand)		R8	HM217395	HM217527	HM217633
R3520	*Rattus exulans*	Sakhon Nakhon (Thailand)	MahiU	R8	HM217424	HM217553	HM217662
R3563	*Rattus exulans*	Surat Thani (Thailand)	KU	R8	HM217428	HM217557	HM217666
R5349	*Rattus exulans*	Nan (Thailand)	CBGP	R8	HM217470	HM217595	HM217703
R5447	*Rattus exulans*	Nan (Thailand)	CBGP	R8	HM217472	HM217596	HM217708

CB0001	*Rattus argentiventer*	Veal Renh (Cambodia)	MahaU	R6	HM217362	HM217484	HM217600
CB0104	*Rattus argentiventer*	Veal Renh (Cambodia)	MahaU	R6	HM217364	HM217486	HM217602

R3087	*Rattus andamanensis*	Kanchanaburi (Thailand)		R7	HM217403	HM217533	HM217641
**R4377**	***Rattus andamanensis***	**Loei (Thailand)**	MahaU	R2	HM217452	HM217579	HM217689
**R3548**	***Rattus andamanensis***	**Phrae (Thailand)**	KU	R2	HM217426	HM217555	HM217664
**R4481**	***Rattus andamanensis***	**Phrae (Thailand)**	MahaU	R2	HM217458	HM217584	HM217695
**R0130**	***Rattus andamanensis***	**Ratchaburi (Thailand)**		R2	HM217371	HM217502	HM217608
**R2976**	***Rattus andamanensis***	**Nakhon Pathom (Thailand)**		**R3**	HM217397	HM217528	HM217635

R3565	*Rattus norvegicus*	Nakhon Pathom (Thailand)	MahiU	R9	HM217429	-	-
R0223	*Rattus norvegicus*	Ratchaburi (Thailand)		R9	HM217373	HM217504	HM217611
R0115	*Rattus norvegicus*	Ratchaburi (Thailand)		R9	HM217370	HM217501	HM217609
RNO 032	*Rattus norvegicus*	Cambodia		R9	HM217481	HM217499	-

L0180	*Rattus nitidus*	Luang Prabang (LPDR)	MahaU	R10	HM217478	HM217492	HM217715
L0192	*Rattus nitidus*	Luang Prabang (LPDR)	MahaU	R10	HM217479	HM217493	HM217716

R4188	*Rattus sp.*	Phrae (Thailand)	CBGP	**R3**	HM217442	HM217569	HM217679
L0010	*Rattus sp.*	Luang Prabang (LPDR)	MahaU	R10	HM217474	HM217488	HM217711
**R0856**	***Bandicota indica***	**Nakhon Pathom (Thailand)**		R8	HM217379	HM217510	HM217617
R4001	*Bandicota indica*	Kalasin (Thailand)	MahaU	B1	HM217435	-	HM217672
R3189	*Bandicota indica*	Kanchanaburi (Thailand)		B1	HM217408	HM217538	HM217646
R4265	*Bandicota indica*	Loei (Thailand)	CBGP	B1	HM217447	HM217574	HM217684
R1006	*Bandicota indica*	Nakhon Ratchasima (Thailand)		B1	HM217380	HM217511	HM217618
R3521	*Bandicota indica*	Phrae (Thailand)	KU	B1	HM217425	HM217554	HM217663
R0269	*Bandicota indica*	Ratchaburi (Thailand)		B1	HM217376	HM217507	HM217614
R0304	*Bandicota indica*	Ratchaburi (Thailand)		B1	HM217378	HM217509	HM217616
R5313	*Bandicota indica*	Nan (Thailand)	MahaU	B1	HM217469	HM217594	HM217706
L0142	*Bandicota indica*	Luang Prabang (LPDR)	MahaU	B1	HM217476	HM217490	HM217713
**R4408**	***Bandicota indica***	**Loei (Thailand)**	CBGP	B2	HM217455	-	HM217692

**R1284**	***Bandicota savilei***	**Nakhon Ratchasima (Thailand)**		B1	HM217386	HM217517	HM217624
**R1822**	***Bandicota savilei***	**Nakhon Pathom (Thailand)**		B1	HM217390	HM217521	HM217628
R1797	*Bandicota savilei*	Kanchanaburi (Thailand)		B2	HM217387	HM217518	HM217625
R1191	*Bandicota savilei*	Nakhon Ratchasima (Thailand)		B2	HM217385	HM217516	HM217623
R3550	*Bandicota savilei*	Phrae (Thailand)	KU	B2	HM217427	HM217556	HM217665

R0093	*Bandicota sp.*	Ratchaburi (Thailand)		B2	HM217369	HM217500	HM217607

R3050	*Berylmys berdmorei*	Kanchanaburi (Thailand)		Be1	HM217401	HM217532	HM217639
R4266	*Berylmys berdmorei*	Loei (Thailand)	CBGP	Be1	HM217448	HM217575	HM217685
R3441	*Berylmys berdmorei*	Loei (Thailand)	MahiU	Be1	HM217418	HM217547	HM217656
R5310	*Berylmys berdmorei*	Nan (Thailand)	MahaU	Be1	HM217468	-	HM217709
L0006	*Berylmys berdmorei*	Luang Prabang (LPDR)	MahaU	Be1	HM217473	HM217487	HM217710
R3618	*Berylmys berdmorei*	Phrae (Thailand)	KU	Be1	HM217432	HM217560	HM217669
R3603	*Berylmys berdmorei*	Phrae (Thailand)	KU	Be1	HM217431	HM217559	HM217668

R4400	*Berylmys bowersi*	Loei (Thailand)	MahaU	Be2, a	HM217453	HM217580	HM217690
R3425	*Berylmys bowersi*	Loei (Thailand)	KU	Be2, a	HM217415	HM217544	HM217653
R3415	*Berylmys bowersi*	Loei (Thailand)	KU	Be2, a	HM217413	HM217542	HM217651
R5410	*Berylmys bowersi*	Nan (Thailand)	MahaU	Be2, a	HM217471	-	HM217707
L0151	*Berylmys bowersi*	Luang Prabang (LPDR)	MahaU	Be2, a	HM217477	HM217491	HM217714
**R3268**	***Berylmys bowersi***	**Kanchanaburi (Thailand)**	KU	Be2, b	HM217412	HM217597	HM217650

**R4098**	***Leopoldamys sabanus***	**Loei (Thailand)**	CBGP	L1	HM217439	HM217566	HM217676
**R4222**	***Leopoldamys sabanus***	**Loei (Thailand)**	MahaU	L1	HM217444	HM217571	HM217681
**R4296**	***Leopoldamys sabanus***	**Phrae (Thailand)**	MahaU	L1	HM217450	HM217577	HM217687
**R4276**	***Leopoldamys sabanus***	**Phrae (Thailand)**	CBGP	L1	HM217449	HM217576	HM217686
**R4370**	***Leopoldamys sabanus***	**Phrae (Thailand)**	CBGP	L1	HM217451	HM217578	HM217688
R3111	*Leopoldamys sabanus*	Kanchanaburi (Thailand)		L3	HM217404	HM217534	HM217642
R3033	*Leopoldamys sabanus*	Kanchanaburi (Thailand)		L3	HM217400	HM217531	HM217638

R4517	*Leopoldamys neilli*	Loei (Thailand)	MahaU	L2	HM217462	HM217588	HM217699
R4527	*Leopoldamys neilli*	Loei (Thailand)	MahaU	L2	HM217463	HM217590	HM217701
R4486	*Leopoldamys neilli*	Phrae (Thailand)	MahaU	L2	HM217460	HM217586	HM217697
R4485	*Leopoldamys neilli*	Phrae (Thailand)	MahaU	L2	HM217459	HM217585	HM217696

R3419	*Leopoldamys sp.*	Loei (Thailand)	KU	L1	HM217414	HM217543	HM217652

R4723	*Niviventer fulvescens*	Loei (Thailand)	MahaU	N1	HM217465	HM217591	HM217702
**R3212**	***Niviventer fulvescens***	**Kanchanaburi (Thailand)**	KU	N2	HM217409	HM217539	HM217647

R4525	*Niviventer sp.*	Loei (Thailand)	MahaU	N1	HM217464	HM217589	HM217700
R3427	*Niviventer sp.*	Loei (Thailand)	KU	N1	HM217416	HM217545	HM217654
R3429	*Niviventer sp.*	Loei (Thailand)	KU	N1	HM217417	HM217546	HM217655
R3459	*Niviventer sp.*	Loei (Thailand)	KU	N1	HM217419	HM217548	HM217657
R4497	*Niviventer sp.*	Phrae (Thailand)	MahaU	N1	HM217461	HM217587	HM217698
R3492	*Niviventer sp.*	Loei (Thailand)	KU	N1	HM217422	HM217551	HM217660
R3077	*Niviventer sp.*	Kanchanaburi (Thailand)	MahiU	N3	HM217402	-	HM217640

R3795	Nu Deng*	Khammouane (LPDR)	MahiU	N4	HM217433	HM217561	HM217670
R3796	Nu Deng*	Khammouane (LPDR)	MahiU	N4	HM217434	HM217562	HM217671

R3118	*Maxomys surifer*	Kanchanaburi (Thailand)		M1	HM217406	HM217536	HM217644
R3116	*Maxomys surifer*	Kanchanaburi (Thailand)		M1	HM217405	HM217535	HM217643
**R4223**	***Maxomys surifer***	**Loei (Thailand)**	CBGP	M2	HM217445	HM217572	HM217682
**R3464**	***Maxomys surifer***	**Loei (Thailand)**	KU	M2	HM217420	HM217549	HM217658

MK0509 BZ02	*Micromys minutus*	China	CBGP	Outgroup	HM217360	HM217482	HM217598
MK0509 BZ07	*Micromys minutus*	China	CBGP	Outgroup	HM217361	HM217483	HM217599

Field identifications were achieved based on morphological criteria according to [33-35] and [11].

"Phylogenetic species" relies on the DNA-based species delimitation method (see also Figure 3).

Mismatches between field identifications and phylogenetic species are highlighted in bold and reflect the difficulty to identify rat species even for experts.

"Nu deng*" was assigned to animal identified but impossible to assigned to a particular species; in Thai language, "red rat".

"-" corresponds to missing data in the phylogenetic analyses.

Voucher locations:

CBGP: Centre de Biologie et de Gestion des Populations, Montpellier, France - curator of the collections, Y. Chaval, chaval@supagro.inra.fr/KU: Kasetsart University, Bangkok, Thailand - curator: W. Rerkamnuaychoke/MahiU: Mahidol University, Nakhon Pathom, Thailand - curator: V. Herbreteau, vincent.herbreteau@cirad.fr/MahaU: Mahasarakham University, Mahasarakham, Thailand - curator: S. Soonchan

See Figure 1 for additional information about sample locations.

For nomenclatural prospects, a small piece of skin from the holotype specimen of *Leopoldamys neilli *was also analysed in this study. The type specimen is the male n°54-4330 from the Centre for Thai National Reference collections, collected by W.A. Neill in 1973 at Wat Tham Prapothisat, in the Saraburi Province (Kaengkhoi District, Thailand, 14°35'N X 101°8'E) (see [33] for further details).

### 2. Sequence acquisition

Three genes proven valuable for rodent systematics were considered for the phylogenetic analyses [39,40,25,17]. We targeted two mitochondrial markers, the cytochrome *b *(cytb) and the cytochrome c oxydase I (COI) genes and the first exon of the nuclear gene encoding the interphotoreceptor retinoid binding protein (IRBP).

To avoid contamination, pre-amplification procedures and post-amplification analyses were performed in independent rooms in the laboratory. DNA was extracted from tissue with DNEasy Tissue Kit (Qiagen) in accordance with the manufacturer's instructions. Primer sets used to amplify the cytb, COI and IRBP genes are listed in Table 2. All amplifications were carried out in 25 μL reactions containing about 30 ng of extracted DNA, 0.2 mg/mL BSA (Roche, 1 mg/mL), 300 μM of each dNTP, 0.2 μM of each primer, 1 unit of Taq polymerase (Qiagen), 2.5 μL of 10X buffer, 0.5 mM of extra MgCl2. Cycling conditions were as follows: one activation step at 94°C for 4 min followed by 40 cycles of denaturation at 94°C for 30 s, annealing at 48°C-58°C depending on the primers (Table 2) for 30 s, elongation at 72°C for 45 s-1'30 min depending on the length of the target (1 minute per kb), and a final extension at 72°C for 10 min. PCR products were sequenced by Macrogen (Seoul, South Korea).

**Table 2:** Primers and PCR cycling conditions used in this study.

Designation	Gene Name	Nucleotide sequence 5' → 3'	Annealing Temperature	Fragment Length (bp)	Original Publication
**Cytb**	**cytochrome *b***				
L14723		ACCAATGACATGAAAAATCATCGTT	50°C	1213	[69]
H15915		TCTCCATTTCTGGTTTACAAGAC			
**COI**	**Cytochrome c oxydase I**				
BatL5310		CCTACTCRGCCATTTTACCTATG	48°C	750	[25]
R6036R		ACTTCTGGGTGTCCAAAGAATCA			
**IRBP1**	**Interphotoreceptor retinoid binding protein (fragment 1)**				
I1-Rattus		ATTGAGCAGGCTATGAAGAG	58°C	785	this study
J2-Rattus		TAGGGCTTGCTCYGCAGG			
**IRBP2**	**Interphotoreceptor retinoid binding protein (fragment 2)**				
I2		ATCCCCTATGTCATCTCCTACYTG	52°C	892	[70]
J1		CGCAGGTCCATGATGAGGTGCTCCGTGTCCTG			
**cytb barcode**	**Cytochrome *b *(museum specimens)**				
MPLeopol-fw MPRattusSL-Rev		GAYAAAATYCCATTCCACCC TARTTRTCYGGGTCTCC	48°C	122	this study

The IRBP gene was amplified into two overlapping fragments, IRBP1 and IRBP2.

### 3. Phylogenetic analyses

Sequences were aligned by eye using SEAVIEW [41] and translated into peptide sequences using the Transeq EMBOSS tool [42] to exclude putative NUMt copies and to ensure sequence orthology. As the risk of homoplasy by convergence and reversal is reduced by considering a large number of characters [43], we combined the three genes into a single dataset using the DAMBE software [44]. Thus, a total of 3,068 bp were considered in the subsequent phylogenetic analyses.

Base composition bias was evaluated using PAUP* v4.0b10 [45], and a chi-square test was performed to check for taxa with deviations of nucleotide composition. Substitutional saturation was assessed via saturation plots. Using DAMBE [44], the absolute number of transitions was plotted against MLComposite TN93 (Tamura-Nei Model) distance for all pairwise comparisons of taxa. For the three genes, the curve did not reach a plateau when subtracting the third codon position, but did reach a plateau when considering the entire sequences (data not shown). To discard fast evolving transitions and improve inferences without drastically compromising the resolution, we decided to recode the third codon position nucleotides to two state categories, R (purine) and Y (pyrimidine), (RY-coding strategy; [46]).

Phylogenetic trees were reconstructed using two probabilistic approaches: maximum likelihood (ML) and Bayesian inferences (BI). The appropriate model of evolution was first determined for each gene and for the concatenated dataset (with and without RY-coding) using corrected Akaike information criterion (AICc) and MrAIC [47]. The HKY+I+Γ model was selected for both the cytb and COI genes while the GTR+ Γ was selected for the IRBP gene and the combined dataset (with and without RY-coding). ML analyses were performed with PhyML-v2.4.4 [48]. For each analysis, the transition/transversion ratio, the proportion of invariable sites as well as the gamma distribution parameter (if necessary) were estimated and the starting tree was determined by BioNJ analysis of the dataset (default settings). Using optimization options, 500 bootstrap (Bp) replicates were performed. PhyML analyses were first run independently on each locus and then on the combined dataset (with and without RY-coding). Taking into account that PhyML does not allow data-partitioning, partitioned ML analysis was also performed using RAxML 7.0.4 [49]. As the model choice is limited in RAxML, the general time-reversible (GTR) + Γ model (option -m GTRGAMMA) was selected for the three partitions (option -q multipleModelFileName), and individual α-shape parameters, GTR-rates and base frequencies were estimated and optimized for each partition. Robustness of the tree was assessed using the rapid bootstrap procedure (option -f a) with 100 replications (option -# numberOfRuns) [50].

Bayesian analyses were performed using MrBayes v3.1 [51]. Four independent runs of 5,000,000 generations each were performed applying appropriate independent models of evolution to each gene. A burn-in period of 1,000,000 generations was determined graphically using Tracer1.2 [52]. For each dataset, all runs gave similar tree topologies and posterior probability (pp) values.

Alternative topologies were finally tested for significance using the Shimodaira-Hasegawa test (SH test) [53] (RELL option, 1000 Bp replicates) in PAUP* v4.0b10 [45].

### 4. Species delimitation: DNA-based species delimitation method

We used the DNA-based approach proposed by Pons *et al. *[28]. Using a likelihood framework, this new procedure detects the switch in the rate of lineage branching of a tree from interspecific long branches to intraspecific short budding branching and identifies clusters of specimens corresponding to putative species. Two models are implemented to account for the branching process of the entire tree. Under the null model, the whole sample derives from a single population obeying a coalescent process. The alternative model, called general mixed Yule coalescent (GMYC) model combines equations that separately describe branching within populations (coalescent process) and branching between species (a Yule model including speciation and extinction rates). Under the GMYC model, a threshold (T) is optimized such that nodes before the threshold are considered as species diversification events, whereas branches crossing the threshold define clusters following a coalescent process. A standard likelihood ratio test (LRT) is used to assess whether the alternative model provides a better fit than the null model. If the GMYC model is favoured over the null model, the T parameter of the maximum likelihood solution allows the number of species to be estimated. This test was achieved using the R code provided by T. G. Barraclough. This latest version outputs the estimates of the number of species, of the threshold time and their 95% confidence limits (*i.e. *solutions with 2-log likelihood units of the maximum).

Because a pre-requisite of the method is an ultrametric tree, we used the relaxed Bayesian dating method implemented in Multidivtime [54] to convert our optimal phylogram tree (estimated from the Bayesian analysis of the combined dataset) in a rooted additive tree with terminal nodes equally distant to the root. In this aim, we followed the documentation files written by Rutschmann [55] and the procedure detailed in [29]. The settings for the Markov chain Monte Carlo analyses were slightly modified (200,000 cycles in which the Markov chain was sampled 20,000 times every 10^th ^cycle following a burnin period of 100,000 cycles). No fossil is described to calibrate our Rattini phylogeny. As our aim was simply to obtain an ultrametric tree, prior ages to lineages were arbitrarily assigned to 1 (rttm = 1; rttmsd = 0). The mean of the prior distribution for the rate of molecular evolution at the ingroup root node (rtrate) was computed as the mean of the median of the amount of evolution for the different tips of the three independent gene trees (rtrate = 0.735; rtratesd = 0.367).

### 5. Species identification

#### 5.1. Within the Rattus genus

*Rattus *cytb (663 bp) and COI (655 bp) sequences obtained by Robins *et al. *[25] were extracted from GenBank and added to our mitochondrial (*mt*) dataset (see Table 3). As our study focuses on rodents from the Indochinese region, sequences of species belonging to the *Rattus fuscipes *species group (*i.e. *native Australian species) and to the *Rattus leucopus *species group (*i.e. *species indigenous to New Guinea and adjacent archipelagos) were not incorporated in this dataset. Two other unpublished cytb sequences of *R. argentiventer *and *R. sikkimensis *(synonym of *R. andamanensis*) provided by O. Verneau and F. Catzeflis were also included in the subsequent analysis. Sequences of a single representative of *Berylmys, Niviventer, Leopoldamys, Maxomys *and *Micromys *were used to root our mitochondrial phylogeny. Therefore, the *mt *dataset included 129 sequences corresponding to 1,318 bp of *mt *DNA. Partitioned ML analysis was performed using RAxML 7.0.4 [49] and the same options as before.

**Table 3:** Sequences from previous studies included in the *mt *dataset.

Voucher	Nominal species	Origin of specimen	Cytb	COI	Phylogenetic species
RrHu1	*R. rattus*	Huahine, Society Islands	[GenBank: EF186469]	[GenBank: EF186584]	R1
RrSamoa2	*R. rattus*	Samoa	[GenBank: EF186475]	[GenBank: EF186590]	R1
RrRa18	*R. rattus*	Raiatea, Society Islands	[GenBank: EF186474]	[GenBank: EF186589]	R1
ABTC50177	*R. rattus*	Sideia Is., Papua New Guinea	[GenBank: EF186472]	[GenBank: EF186587]	R1

**ABTC64906**	***R. rattus diardi *(1)**	**Kuala Lumpur, Malaysia**	[GenBank: EF186413]	[GenBank: EF186528]	**R3**
**ABTC64907**	***R. rattus diardi***	**Kuala Lumpur, Malaysia**	[GenBank: EF186409]	[GenBank: EF186524]	**R3**
**ABTC64908**	***R. rattus diardi***	**Kuala Lumpur, Malaysia**	[GenBank: EF186410]	[GenBank: EF186525]	**R3**
**ABTC64909**	***R. rattus diardi***	**Kuala Lumpur, Malaysia**	[GenBank: EF186411]	[GenBank: EF186526]	**R3**
**ABTC64910**	***R. rattus diardi***	**Kuala Lumpur, Malaysia**	[GenBank: EF186412]	[GenBank: EF186527]	**R3**

**ABTC 8529**	***R. kandianus *(2)**	**Sri Lanka**	[GenBank: EF186444]	[GenBank: EF18655]	**R3**
**ABTC 8536**	***R. kandianus***	**Sri Lanka**	[GenBank: EF186445]	[GenBank: EF186560]	**R3**
**ABTC 8540**	***R. kandianus***	**Sri Lanka**	[GenBank: EF186446]	[GenBank: EF186561]	**R3**

ABTC 8487	*R. tanezumi*	Amami Island, Japan	[GenBank: EF186508]	[GenBank: EF186623]	R2
ABTC 8562	*R. tanezumi*	Amami Island, Japan	[GenBank: EF186510]	[GenBank: EF186625]	R2
ABTC47981	*R. tanezumi*	Yogyakarta, Indonesia	[GenBank: EF186493]	[GenBank: EF186608]	R2
ABTC47982	*R. tanezumi*	Yogyakarta, Indonesia	[GenBank: EF186494]	[GenBank: EF186609]	R2
ABTC47983	*R. tanezumi*	Yogyakarta, Indonesia	[GenBank: EF186495]	[GenBank: EF186610]	R2
ABTC47984	*R. tanezumi*	Yogyakarta, Indonesia	[GenBank: EF186502]	[GenBank: EF186617]	R2
ABTC47985	*R. tanezumi*	Yogyakarta, Indonesia	[GenBank: EF186503]	[GenBank: EF186618]	R2
ABTC47986	*R. tanezumi*	Yogyakarta, Indonesia	[GenBank: EF186504]	[GenBank: EF186619]	R2
ABTC47987	*R. tanezumi*	Yogyakarta, Indonesia	[GenBank: EF186505]	[GenBank: EF186620]	R2
**ABTC47988**	***R. tanezumi***	**Yogyakarta, Indonesia**	[GenBank: EF186506]	[GenBank: EF186621]	**R3**
ABTC47989	*R. tanezumi*	Yogyakarta, Indonesia	[GenBank: EF186507]	[GenBank: EF186622]	R2
**ABTC47992**	***R. tanezumi***	**Jakarta, Indonesia**	[GenBank: EF186490]	[GenBank: EF186605]	**R3**
ABTC47993	*R. tanezumi*	Jakarta, Indonesia	[GenBank: EF186491]	[GenBank: EF186606]	R2
**ABTC47994**	***R. tanezumi***	**Jakarta, Indonesia**	[GenBank: EF186492]	[GenBank: EF186607]	**R5**
**ABTC47995**	***R. tanezumi***	**Jakarta, Indonesia**	[GenBank: EF186496]	[GenBank: EF186611]	**R3**
**ABTC47996**	***R. tanezumi***	**Jakarta, Indonesia**	[GenBank: EF186497]	[GenBank: EF186612]	**R3**
**ABTC47997**	***R. tanezumi***	**Jakarta, Indonesia**	[GenBank: EF186498]	[GenBank: EF186613]	**R3**
**ABTC47998**	***R. tanezumi***	**Jakarta, Indonesia**	[GenBank: EF186499]	[GenBank: EF186614]	**R3**
**ABTC47999**	***R. tanezumi***	**Jakarta, Indonesia**	[GenBank: EF186500]	[GenBank: EF186615]	**R3**
**ABTC48000**	***R. tanezumi***	**Jakarta, Indonesia**	[GenBank: EF186501]	[GenBank: EF186616]	**R3**
**ABTC48004**	***R. tanezumi***	**Northern Sulawesi, Indonesia**	[GenBank: EF186511]	[GenBank: EF186626]	**R3**
**ABTC48005**	***R. tanezumi***	**Northern Sulawesi, Indonesia**	[GenBank: EF186512]	[GenBank: EF186627]	**R3**
ABTC 8489	*R. flavipectus ***(3)**	Hong Kong, China	[GenBank: EF186440]	[GenBank: EF186555]	R2

Chat2	*R. exulans*	Chatham Islands, New Zealand	[GenBank: EF186426]	[GenBank: EF186541]	R8
CI 6	*R. exulans*	Aitutaki, Cook Islands	[GenBank: EF186414]	[GenBank: EF186529]	R8
Fiji1	*R. exulans*	Fiji	[GenBank: EF186417]	[GenBank: EF186532]	R8
Hawaii3	*R. exulans*	Hawaii	[GenBank: EF186418]	[GenBank: EF186533]	R8
Hu38	*R. exulans*	Huahine, Society Islands	[GenBank: EF186420]	[GenBank: EF186535]	R8
Kap6	*R. exulans*	Kapiti Island, New Zealand	[GenBank: EF186425]	[GenBank: EF186540]	R8
Ra22	*R. exulans*	Raiatea, Society Islands	[GenBank: EF186429]	[GenBank: EF186544]	R8
RNZAwa01	*R. exulans*	Great Barrier Island, New Zealand	[GenBank: EF186424]	[GenBank: EF186539]	R8
Samoa 3	*R. exulans*	Manua, Samoa	[GenBank: EF186430]	[GenBank: EF186545]	R8
Taku5	*R. exulans*	Takutea, Cook Islands	[GenBank: EF186416]	[GenBank: EF186531]	R8
UaHuka4	*R. exulans*	UaHuka, Marquesas Islands	[GenBank: EF186422]	[GenBank: EF186537]	R8
ABTC 8480	*R. exulans*	Thailand	[GenBank: EF186434]	[GenBank: EF186549]	R8
ABTC 8553	*R. exulans*	Thailand	[GenBank: EF186432]	[GenBank: EF186547]	R8
ABTC 8559	*R. exulans*	Thailand	[GenBank: EF186433]	[GenBank: EF186548]	R8
ABTC43078	*R. exulans*	Yuro, Papua New Guinea	[GenBank: EF186427]	[GenBank: EF186542]	R8
ABTC48011	*R. exulans*	Cibodas Forest, Java, Indonesia	[GenBank: EF186421]	[GenBank: EF186536]	R8
ABTC48895	*R. exulans*	Nagada Harbour, Papua New Guinea	[GenBank: EF186428]	[GenBank: EF186543]	R8
ABTC65753	*R. hoffmanni*	Tangoa, Sulawesi, Indonesia	[GenBank: EF186443]	[GenBank: EF186558]	-
ABTC65754	*R. hoffmanni*	Tangoa, Sulawesi, Indonesia	[GenBank: EF186441]	[GenBank: EF186556]	-
ABTC65809	*R. hoffmanni*	Mt Nokilalaki, Sulawesi, Indonesia	[GenBank: EF186442]	[GenBank: EF186557]	-

Rargen_1266	R. argentiventer**	Bangkok, Thailand	O.Verneau, unpublished	-	R6

Rsikki_866	R. sikkimensis **(4)	Mocchan, Vietnam	O.Verneau, unpublished	-	R7

ABTC48025	*R. tiomanicus*	Cibodas Forest, Java, Indonesia	[GenBank: EF186514]	[GenBank: EF186629]	R5
ABTC48026	*R. tiomanicus*	Cibodas Forest, Java, Indonesia	[GenBank: EF186513]	[GenBank: EF186628]	R5

Rn Ra 15	*R. norvegicus*	Raiatea, Society Islands	[GenBank: EF186462]	[GenBank: EF186577]	R9
Rn Hu 21	*R. norvegicus*	Huahine, Society Islands	[GenBank: EF186461]	[GenBank: EF186576]	R9

"Nominal species" stands for the identification given to the specimen by the curator or the collector ([25] and F. Catzeflis, pers. comm.).

"Phylogenetic species" relies on the DNA-based species delimitation method (see also Figure 3).

(1) *Rattus rattus diardi*: Robins et al [25] reports that the specimens ABTC64906-64910 are identified by the South Australian Museum as the subspecies *Rattus rattus diardi *(not *diardii*) as listed by Ellerman [71] on the basis on *R. r. diardi *after Jentink [72]. As already mentioned by Robins et al., [25], *R. diardii *(after Jentink 1880) is however considered as a synonym for *R. tanezumi *by Musser and Carleton [16] but there is no 1880 reference in their bibliography.

(2) *R. kandianus *is listed as a synonym of *R. rattus *[16], (3) *R. flavipectus *of *R. tanezumi *[16], (4) *R. sikkimensis *of *R. andamanensis *[16].

** indicates that specimens are no more available in the mammal tissue collection housed at the Institut des Sciences de l'Evolution de Montpellier [73].

Mismatches between nominal species and phylogenetic species are highlighted in bold.

#### 5.2. Ancient DNA analysis of a holotype specimen

For species assignment, we tested the relevance of DNA sequences obtained from a holotype specimen. As museum samples contain tiny amounts of poorly preserved DNA, we selected a 85 bp fragment of the cytb gene, corresponding to positions from 666 to 750 of the gene sequence of *Rattus norvegicus *(NCBI accession number [GenBank NC_001665]). This fragment was chosen for the following reasons: i) it corresponds to an highly variable region of the gene that allows the discrimination of most vertebrate species including the closest related ones [56] ii) its short length is suited for the PCR amplification of degraded DNA [56] and iii) it has proved valuable for species assignment based on degraded DNA extracted from archaeological samples [57].

To check if it provides adequate discrimination for rat species, the whole cytb sequences of the 122 specimens were reduced to the 85 bp fragment following the groups evidenced by the DNA-based species delimitation method. Based on our sampling, rat species could be easily discriminated with this small sequence (except the two entities hereafter named Be2a and Be2b but see discussion) (see the 85 bp alignment in additional file 1). So, we decided to target this DNA barcode from the holotype of *Leopoldamys neilli*.

As we used a museum specimen, the difficulties associated with ancient DNA studies are relevant to this analysis. Hence, ancient DNA work was performed at the PALGENE national platform (CNRS, ENS Lyon, France) dedicated to ancient DNA analysis, following the standard procedures and using specific equipment and personal protections [58,59].

DNA was extracted from the holotype of *Leopoldamys neilli *following the protocol detailed by Rohland and Hofreiter [60]. Primer sets declined from Télétchéa *et al.*, [56] were used for PCR attempts (Table 2). At least two independent PCR amplifications were performed in 25 μL reaction volumes containing 2.5 units of Perkin Elmer Gold *Taq *polymerase (Applied Biosystems), 1 mg/mL BSA (Roche, 20 mg/mL), 2 mM MgCl_2_, 250 μM of each dNTP, 0.5 μM of primers. For each independent PCR attempt, a range of dilutions was performed to find the best compromise between inhibitor's concentration and targeted DNA molecule concentration. DNA was amplified with a 5 min activation step at 95°C followed by 55 cycles of denaturation (94°C, 30 s), annealing (48°C, 30 s) and elongation (72°C, 45 s). Amplification products were systematically cloned using Topo TA Cloning for sequencing kit (Invitrogen). 16 clones of independent amplifications were sequenced to determine the consensus sequence (Macrogen, Seoul, South Korea).

The CAOS software, a two step character-based DNA barcoding method [61] was then used to determine if the *Leopoldamys neilli *holotype consensus sequence could be assigned to one of the clusters recognized as a putative species by the method of Pons *et al.*, [28]. First, a diagnostic rules generator, P-Gnome, was used to search DNA changes through the 85 bp cytb matrix (122 sequences) and to establish diagnostic rule sets for each of the previously described entities (outputs of the DNA-based species delimitation method). Then, the P-Elf program was run to classify as a query the holotype sequence according to the rules generated by P-Gnome.

## Results

### 1. Sequence analyses and phylogenetic reconstructions

Cytb, IRBP and COI sequences were generated for 122, 120 and 116 rat specimens respectively. All sequences were deposited in GenBank under the accession numbers HM217360 to HM217717 (Table 1). No significant difference in nucleotide composition among taxa was detected which indicated that no artificial grouping could occur due to a misleading compositional signal in the dataset. PhyML analyses were first carried out on each locus independently (data not shown). Each gene considered separately does not result in a robust Rattini phylogeny: mitochondrial markers help to resolve terminal nodes, while IRBP lends support to deepest ones. But, since the 3 genes yielded consistent, compatible topologies, sequences were concatenated and phylogenetic analyses were then carried out using the combined dataset.

Identical topologies were obtained with and without a RY-coding of the 3^rd ^codon position (data not shown). However, better resolution and stronger topological supports (Bp and pp) were reached without an RY recoding strategy. It seems that our dataset was not informative enough for a RY recoding strategy resulting in this case in an over-depletion of the phylogenetic signal.

**Figure 2:**
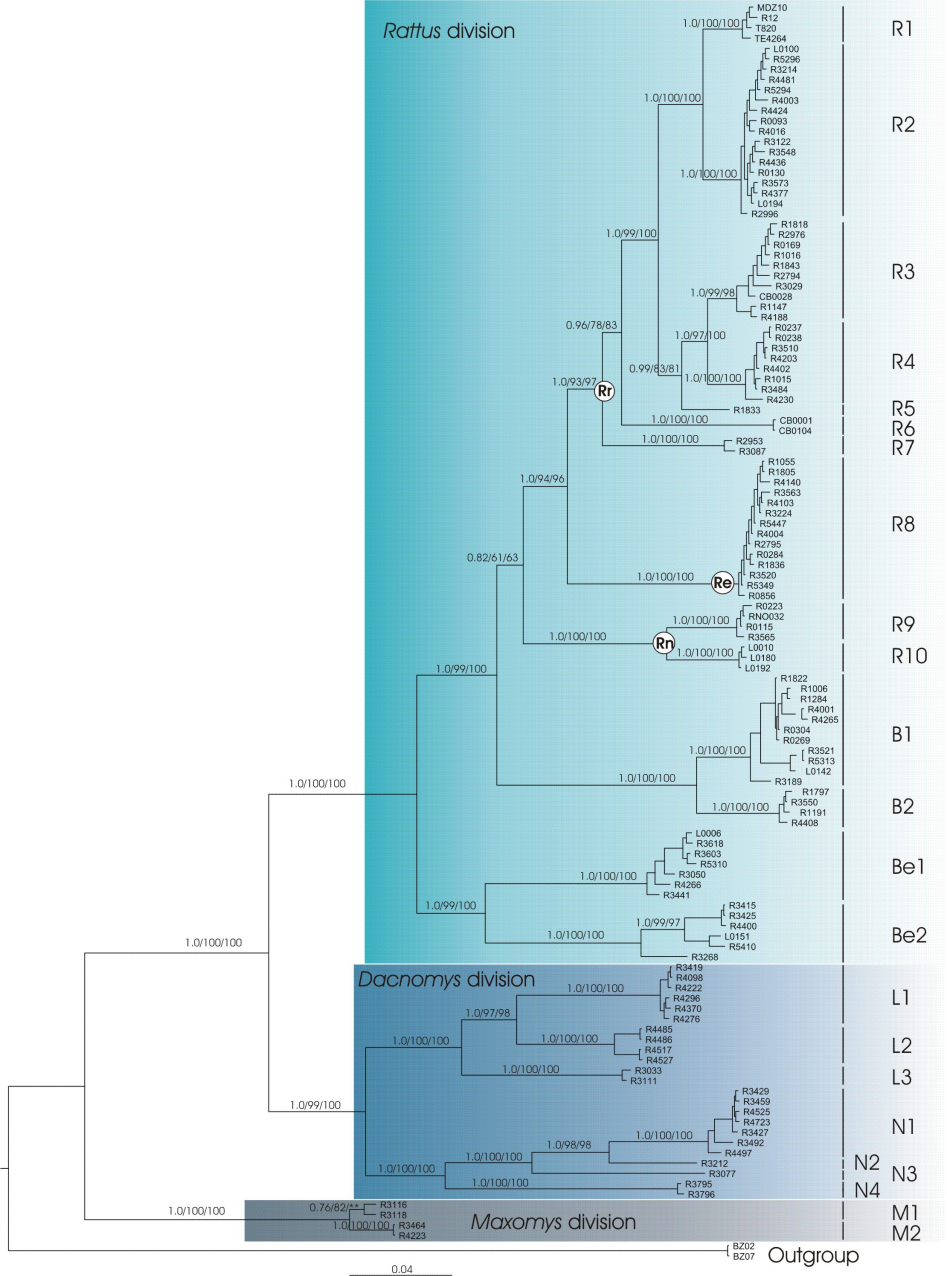
**Phylogenetic tree depicting relationships of the Indochinese Rattini based on the analyses of the combined cytb, COI and IRBP genes and reconstructed following Bayesian method**. BI and ML analyses of the dataset gave an identical topology. Numbers above the branches reflect support obtained from the analysis of the dataset following 3 different reconstruction methods: BI/unpartitioned ML/partitioned ML. Support values are not shown for very short branches. The symbol "**" indicates that phylogenetic relationships are not supported by the partitioned ML analysis. **Rr **stands for *Rattus rattus *species group, **Re **for *Rattus exulans *species group, **Rn **for *Rattus norvegicus *species group, following Musser and Carleton's denominations [16]. At the right hand of the tree, lineages are labelled according to the genus to which they belong.

BI, partitioned and unpartitioned ML analyses (without RY recoding strategy) yielded the identical topology given in Figure 2. Most relationships among the Rattini tribe were well resolved (supports 61-100 for Bp, 0.82-1.00 for pp). Monophyletic groups corresponding to the Rattini divisions proposed by Musser and Carleton [16] are sustained with the highest values of Bp or pp. The *Maxomys *division clearly appears as the first division to diverge followed by the *Dacnomys *division, here represented by *Leopoldamys *and *Niviventer *genera, and the *Rattus division*. *Berylmys *appears with maximum support values as the earliest lineage to diverge among the *Rattus *division. A sister grouping is indicated between the genera *Bandicota *and *Rattus*, but this association is weakly supported. In fact, the monophyly of the *Rattus *genus received moderate pp (0.82) to weak Bp supports (61 for unpartitioned, 63 for partitioned ML analyses). To test the reliability of these findings, we considered an alternative hypothesis concerning the position of *Bandicota *within the *Rattus *division (*i.e. Bandicota *was placed inside the *Rattus sp. *cluster). SH-test failed to find significant differences between these hypotheses and the alternative branching orders of *Bandicota *inside the *Rattus *division could not be excluded (P > 0.05). Inside the *Rattus sp. *clade, the 3 *Rattus *species groups proposed by Musser and Carleton [16] could be distinguished. The *R. exulans *monotypic group (*Re*, Figure 2) clustered with the *R. rattus *species group (*Rr*, Figure 2) with high branch supports (Bp = 94/96 for the unpartitioned/partitioned ML analyses; pp = 1) and the *R. norvegicus *species group (*Rn*, Figure 2) is placed as sister taxa to the *R. exulans *species group/*R. rattus *species group cluster.

At this point in the analysis, 23 lineages (labelled R1 to M2 in the Figure 2) are identified within our taxon sampling. As their specific status are still questioned, intra-generic relationships are problematic to describe and will not be discussed in this section.

### 2. Species delimitation

**Figure 3:**
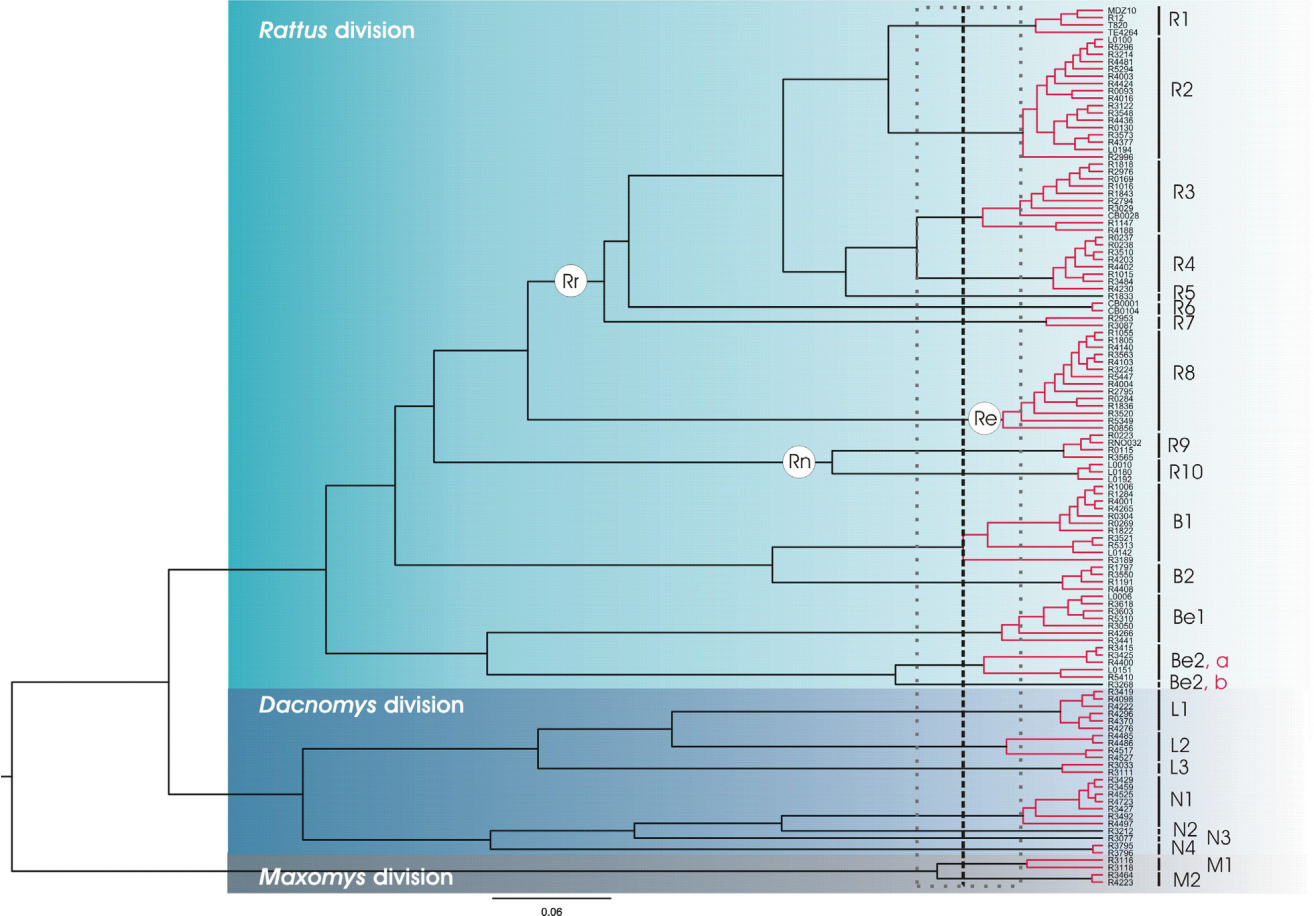
**Rattini ultrametric tree obtained with Multidivtime and clusters of specimens recognized as putative species by the method of Pons *et al. ***[28]. Genetic clusters recognized as a putative species are highlighted in red and separated by longer black branches. The vertical bars group all sequences within each significant cluster, labelled R1 to M2 according to the genus to which they belong. **Rr **for *Rattus rattus *species group, **Re **for *Rattus exulans *species group, **Rn **for *Rattus norvegicus *species group.

The existence of distinct phylogenetic lineages was corroborated by the analysis of the branching rate pattern. A lineage-through-time plot based on the Multidivtime ultrametric tree evidenced a sudden increase in branching rate towards the present, likely corresponding to the switch from interspecies to intraspecies branching events (see additional file 2). To fit the position of the switch, the method of Pons *et al. *[28] was applied to the time calibrated tree (Figure 3). The GMYC model was preferred over the null model of uniform branching rates (*log*L = 700.133, compared to null model *log*L = 687.218; 2ΔL= 25.83, χ2 test, d.f. = 3, p < 0.0001). The model fitted the switch in the branching pattern occurring at -0.07084 (*i.e. *T of the ML solution/it is worth reminding that the time separating the ingroup root from the present was arbitrarily assigned to 1), leading to an estimate of 24 putative species, 4 of which containing a single individual (labelled R5, Be2b, N2 and N3 respectively in Figure 3). Two *Maxomys *(M1 and M2), 4 *Niviventer *(N1 to N4), 3 *Leopoldamys *(L1 to L3), 2 *Bandicota *(B1 and B2), 3 *Berylmys *(Be1, Be2a, Be2b) and 10 *Rattus *species (R1 to R10) could be numbered as indicated in Figure 3. It is worth noting that the *Berylmys *lineage (labelled Be2 in Figure 2) actually seems to correspond to two putative species following Pons et al's approach (therefore labelled Be2a and Be2b in Figure 3). Confidence interval for the threshold ranged from -0.09439 to -0.04189 and the estimated number of species ranged from 22 to 32 (*i.e. *estimates falling within 2 log-likelihood units of the ML solution).

### 3. Species identification

#### 3.1. Within the Rattus genus

**Figure 4:**
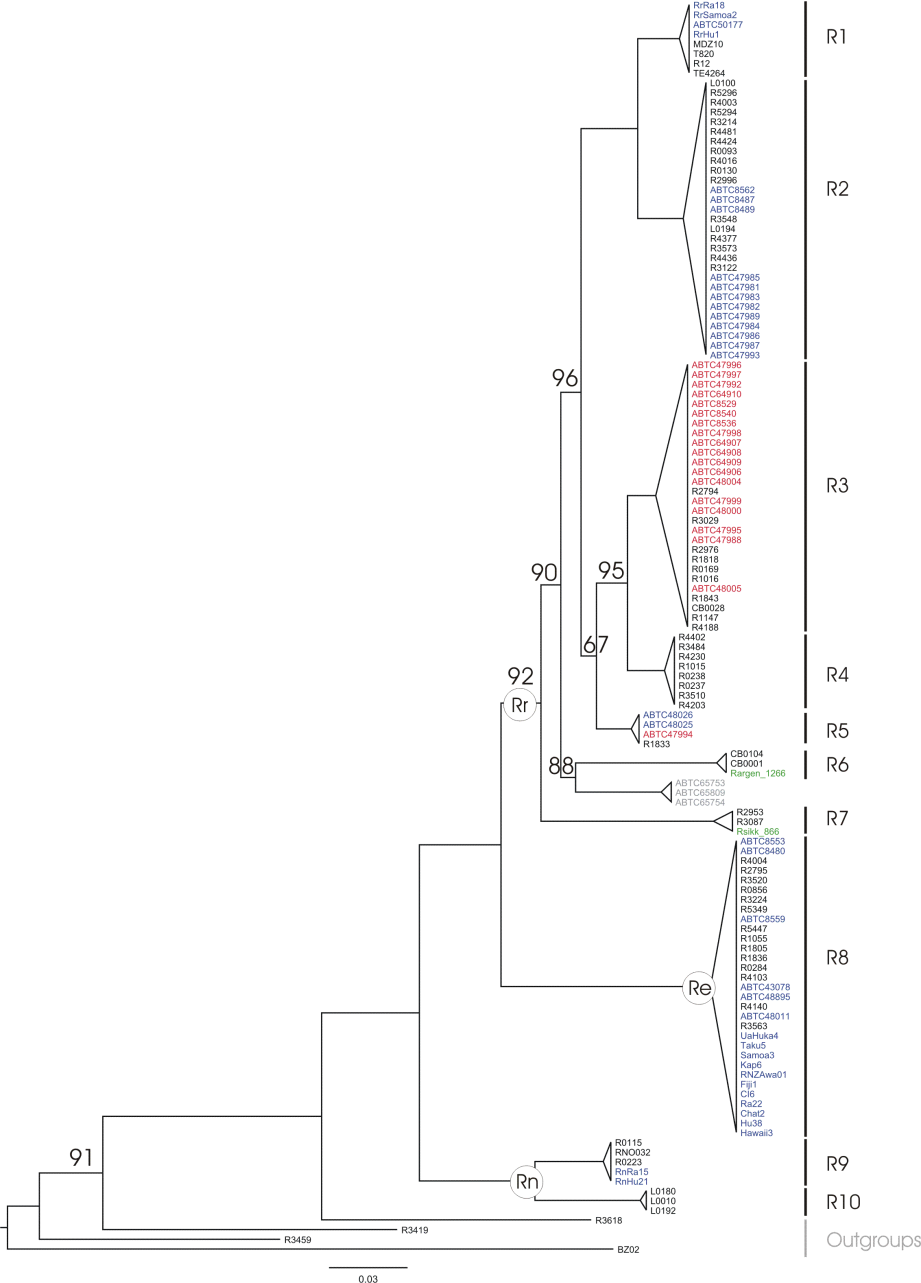
**ML tree depicting relationships within the *Rattus *division on the basis of *mt *dataset and estimated using partitioned ML analysis**. Bp values are shown above branches. Bp values equal to 100% are not indicated. Robins' sequences are highlighted in blue when nominal and phylogenetic species are congruent, in red on the contrary (see also Table 3). *Rattus hoffmanni *sequences are indicated in grey; sequences provided by Verneau and Catzeflis in green. **Rr **for *Rattus rattus *species group, **Re **for *Rattus exulans *species group, **Rn **for *Rattus norvegicus *species group. At the right hand of the tree, cluster denomination is the same as in the Figure 3.

The partitioned ML analysis of the *mt *dataset including 64 new *Rattus *sequences (this study) plus 61 from previous studies [25] gave the highly resolved and robust tree represented in Figure 4. This has allowed us to name some clusters identified as putative species by the DNA-based species delimitation method. Because the monophyly of each cluster embracing the supplementary published sequences is supported with the highest Bp value, the level of confidence of these identifications could be considered as maximal if the voucher identification beforehand is correct.

Robins' sequences identified as *Rattus rattus *cluster with 100% Bp support with sequences assigned to *R. rattus *specimens in [36]. Specific identification of group R1 as *Rattus rattus *is thus convincingly confirmed. According to the *mt *tree, none of our samples from Thailand, Cambodia or Lao PDR could be assigned to this species. Following the same approach, R2 seems to correspond to *Rattus tanezumi*, R5 to *Rattus tiomanicus*, R8 to *Rattus exulans *and R9 to *Rattus norvegicus*. Sequences provided by O. Verneau and F. Catzeflis allow us identifying R6 as *R. argentiventer *and R7 as *R. andamanensis*. As expected, since its distribution is restricted to Sulawesi, sequences of *Rattus hoffmanni *group with none of our specimens. *R. hoffmanni *whose phylogenetic affinities among the *Rattus rattus *group need to be elucidated [16] appears as the sister taxa to *R. argentiventer *with strong support (88 Bp). The situation appears more complex for the species R3. This group corresponds to a mix of specimens identified as *R. rattus diardi *in [25], *Rattus kandianus *(considered as a synonym of *R. rattus*, [16]) in [25], *R. tanezumi *from Indonesia [25] and *R. tanezumi*, *R. andamanensis *or *R. argentiventer *according to the field names we assigned during our sampling. Consequently, no nominal species could be reliably assigned to R3.

According to morphological criteria and because its sistership with *Rattus norvegicus *[16] (see Table 4 in discussion), R10 could be convincingly assigned to *Rattus nitidus*.

**Table 4:** Species names proposed for each species recognized as putative ones by the method of Pons *et al*., [28].

Phylogenetic species	Species name proposed	Phylogenetic evidences	Morphological, geographical and ecological evidences
**R1**	***Rattus rattus***	R1 specimens identified in [36] cluster unambiguously with *R. rattus *specimens identified by Robins *et al. *[25] (see Figure 4). It is worth noting that, during this study, this species was never sampled in the fields in Thailand, Laos and Cambodia.	

**R2**	***Rattus tanezumi***	R2 specimens cluster unambiguously with *R. tanezumi *specimens identified in [25] (see Figure 4).	Medium-sized rat; fur light brown to reddish brown above, white below; dark tail, equal or longer than head and body length; caught in a large range of habitats, from houses, gardens, crops and rice fields to the edge of secondary forests.

**R3**	***Rattus sp.(to be named)***	R3 includes specimens identified as *Rattus diardii *in the study of Robins *et al*., [25] and rats referred to Malaysian house rat (*i.e. Rattus diardii*) by local populations in Indonesia (Andru, J., *pers. comm*.). Today, *Rattus diardii *has been placed as a synonym of *Rattus tanezumi *according to morphological criteria.	Urban rat or rat living near human habitations. Misidentified by us as *Rattus tanezumi*, *R argentiventer *and *R. andamanensis *in the *Rattus rattus *species group.

**R4**	***Rattus losea or "losea-like"***		Medium-sized rat; shaggy fur brownish grey above, white to geyish below; dark tail, shorter than head and body length; caught mostly in rice fields and sometimes in dry agricultural fields. According to Aplin [35] two distinct forms of *R. losea *may exist. True *R. losea *(described from Taiwan) would be distributed from Southern China to central Vietnam. The second form "losea-like" would inhabit the Mekong Delta region from Southern Vietnam, Cambodia, Thailand, to the North of Vientiane Province in Laos. Since our analyses did not include samples from the two putative groups, it was not possible to determine if they are genetically distinct. Until this taxonomic issue is resolved, we prefer to name R4 "*losea*-like".

**R5**	***Rattus tiomanicus***	R5 specimens cluster unambiguously with *R. tiomanicus *specimens identified in [25] (see Figure 4).	Medium-sized rat; fur brown above, white below; dark tail, slightly longer than head and body length; arboreal; caught in palm plantations. Morphologically very similar to *Rattus tanezumi *but with shorter guard hairs.

**R6**	***Rattus argentiventer***	R6 sequences cluster unambiguously with *R. argentiventer *sequences provided by O. Verneau and F. Catzeflis (see Figure 4)/identification of Verneau's specimen confirmed by G. Musser [64].	Medium-sized rat; fur yellowish brown above, grey-white below, with developed guard hair on the back, distinct orange fringe of fur just forward of the ear; dark tail, shorter than head and body length; caught in rice fields and plantations.

**R7**	***Rattus andamanensis***	R7 sequences cluster unambiguously with *R. sikkimensis *sequences provided by O. Verneau and F. Catzeflis (see Figure 4).	Medium-sized rat; fur orange brown above, white-creamy below, with very elongated guard hairs; dark tail, longer than head and body length; caught in evergreen forests.

**R8**	***Rattus exulans***	R8 specimens cluster unambiguously with *R. exulans *specimens identified in [25] (see Figure 4).	Small-sized rat; fur grey-brown above, pale grey below; dark tail, longer than head and body length; domestic species found in houses.

**R9**	***Rattus norvegicus***	R9 specimens cluster unambiguously with *R. norvegicus *specimens identified in [25] (see Figure 4).	Large-sized rat; fur dark-grey above, pale grey below; tail shorter than head and body length, dark above and paler beneath but not clearly separated; occurs in major ports and neighbouring cities.

**R10**	***Rattus nitidus***	Sister relationship with *Rattus norvegicus *evidenced by molecular data (see Figure 2) .	Medium-size rat with a soft woolly fur, dorsally brown and grey-based cream on belly. Pearly white feet. A *nitidus/norvegicus *sistership was proposed by morphologists. According to Musser and Carleton [11], both have "dense and soft fur, six pairs of teats, and an upper M1 in which the anterolabial cusp on the anterior lamina is missing or undetectable due to its coalesence with the adjacent central cusp".

**B1**	***Bandicota indica***	Only two *Bandicota *species have been described in the Indochinese region. Usually, *B. indica *specimens are unambiguously larger than *B. savilei. *Adult *B. savilei *and juvenile or immature *B. indica *may be confounded. A molecular test based on PCR amplifications with specific primers allowing discriminating between the 2 species (Chaval *et al.*, in prep.) was used in such cases (data not shown).	Large-sized rat; fur dark above, grey below; tail shorter than head and body; aggressive and stocky; inhabits agricultural fields. The ratio of pes length to head+body length is used to distinguish *B.indica *from *B.savilei *[74].

**B2**	***Bandicota savilei***		Medium-sized rat; fur dark above, grey below; tail shorter than head and body; inhabits dry lands, grasslands, clearings in forest.

**Be1**	***Berylmys berdmorei***		Medium-sized rat; fur grey above, white below; tail shorter than head and body; inhabits secondary forests and fields close to forests.

**Be2a**	***Berylmys bowersi***		Large-sized rat; fur grey above, white below; tail slightly longer than head and body; inhabits secondary forests and fields close to forests.

**Be2b**	***Berylmys sp.***		*Berylmys mackensiei *has been described in the Indochinese region by Marshall [33]. However the skull of *B. mackenziei *he studied was identified by Musser and Newcomb [75] as *B. bowersi*. Populations of *Berylmys bowersi *in peninsular Thailand were reported to be geographically isolated and to differ in some ways from those elsewhere (here speculated as to be Be2,a) [67]. Be2b specimen came from the Kanchanaburi locality, North to the isthmus of Kra and could consequently belong to this former particular population. Because of the lack of additional information about this specimen, no species name could be convincingly assigned to Be2b.

**L1**	***Leopoldamys edwardsi***		Large-sized rat; fur red-brown above, white-cream below; very long tail, longer than head and body; inhabits secondary forests.

**L2**	***Leopoldamys neilli***	Genuine sequence obtained from the holotype specimen of *L. neilli *was assigned to L2 without ambiguities.	Large-sized rat (but the smallest *Leopoldamys *species); fur greyish -brown above, white-cream below; tail longer than head and body. Until now, the species has been recorded from a few locations in limestone areas of northern and South western Thailand, North of the peninsular region [76]. Our specimens were also trapped on tower karst in northern and northeastern Thailand (Phrae and Loei provinces).

**L3**	***Leopoldamys sabanus***		Large-sized rat; fur red-brown above, white-cream below; very long tail, longer than head and body; inhabits secondary forests. Caught in secondary forests. Often misidentified as *Leopoldamys edwardsi*. The two species of *Leopoldamys sabanus *and *Leopoldamys edwardsi *are indeed morphologically very similar. The species name we proposed for L3 is based on geographical evidences from Marshall (1977). Based on his work, the only *Leopoldamys *species that has been described in Kanchanaburi province is *Leopoldamys sabanus*. The L3 specimens were caught in this province.

**N1**	***Niviventer fulvescens***		Medium-sized rat; spiny fur red-brown above, white-cream below; tail longer than head and body, sharply bicoloured from base to tip; absence of terminal pencil and smallest length of bulla make us exclude *Niviventer confucianus *as species name.

**N2**	***Niviventer sp. 1***		Marshall [67], Musser [77] and Corbet [34] documented the occurrence of *Niviventer bukit *in Kanchanaburi, where representatives of N2 and N3 species were caught. One of the two could be *N. bukit*. However, *bukit *is today considered as conspecific with *Niviventer fulvescens *[16]. Consequently, we prefer to refrain from giving a species name to these 2 species.

**N3**	***Niviventer sp. 2***		

**N4**	***Niviventer langbianis or Chiromyscus chiropus***	N4 is placed at the base of the *Niviventer *group. It could thus belong to the genus *Niviventer *or to a sister genus to *Niviventer. *According to Musser and Carleton [16], *Chiromyscus *is presumed to be one of the closest phylogenetic relatives of *Niviventer. *Based on morphological criteria, this specimen could be a *Chiromyscus chiropus *representative. However, *Chiromyscus chiropus *is morphologically very closed to *N. langbianis*. Thus, N4 could be one of these two species. At the end of this work, we have just received *N. langbianis *samples from the AMCC. Our preliminary work based on mitochondrial DNA suggests that N4 may be *N. langbianis *rather than *C. chiropus*.	Identified in the field as Nu-deng because of its reddish fur (in Lao, "red rat"). Further considerations of pictures of one of the two specimens included in this study show that legs, feet and head are buffy orange as described by Musser [77] regarding *Chiromyscus chiropus*. However, the wide dark brown rings around the eyes are not visible and the tail is not bicoloured as expected for *Chiromyscus. Chiromyscus *is morphologically very close to *Niviventer langbianis *[77] and easily confused with it. Other criteria to discriminate between the two species such as the presence of a nail on each hallux instead of a claw for *Chiromyscus *are not obvious on our pictures. Morphological identification is thus questionable. However, molecular data are tipping the balance for *N. langbianis *assignation.

**M1**	***Maxomys sp.***		Identified by us as *Maxomys surifer *in the field. Could be assigned to *Maxomys rajah *but this species has never been reported in this area. This result could be to a bias of the branching-length method that could have some difficulties to deal with strong phylogeographic pattern. The phylogeography of *Maxomys surifer *was investigated using *mt *DNA but focusing on the large Sunda shelf area [78]. A structuration between the North-eastern Vietnam and the Southern Vietnam seems to exist but this finding is based on only four sampled (for which sequences are not available in databanks). As a greater sampling and more additional data are needed to assess the phylogeographic pattern of this species, we prefer to refrain from giving a species name to this cluster.

**M2**	***Maxomys surifer.***		Medium-sized rat; spiny fur red-brown above, white-cream below; tail slightly longer but nearly equal to head and body length, sharply bicoloured with a white tip. This is the only *Maxomys *species described in this area

The congruence between geographical, morphological and phylogenetic data allows us proposing species names. Waiting for a complete taxonomic revision of the Rattini tribe, these propositions are not definitive but are revisable ones.

#### 3.2 Ancient DNA analysis of a holotype specimen

##### Sequences obtained from holotype specimen

We successfully obtained 85 bp cytb sequences from the *Leopoldamys neilli *holotype. At least two independent PCR runs were performed, positive PCR products were cloned and consensus sequences were determined using clone sequences of independent PCR amplifications. Analysis of the differences observed between the clone sequences and consensus sequence shows that 75% of the degradation was due to deamination of cytosines, as expected from ancient DNA substrates [62,63].

##### Holotype sequence authentication

The consensus sequence was identified as a rat cytochrome *b *sequence using a BLAST program (no *Leopoldamys neilli *cytochrome *b *sequence was available in databanks such as EMBL or GenBank before this study). This sequence is a genuine holotype sequence for the following reasons: (i) Rattini samples were never introduced in the ancient DNA facilities before the analysis of this specimen was performed; (ii) all the 16 clones analysed were identified as rat; (iii) the errors induced by DNA damage are perfectly consistent with the pattern generally observed for ancient DNA sequences (strong bias toward type 2 transitions caused by deamination of cytosine [62,63]); (iv) for each amplification, all three PCR blanks remained negative [58]; (v) independent PCRs were performed and furnished the same conclusions. All in all, these points satisfy criteria of authentication for the ancient DNA work [59].

##### Assignment of the holotype sequence to a cluster

The genuine holotype sequence was deposited in GenBank under the accession number HM235947. It was assigned using the CAOS software to the monophyletic cluster corresponding to the *Leopoldamys *species, L2, in our tree (Figures 2 and 3). Consequently, this monophyletic cluster recognized as a putative species by the method of Pons *et al. *[28] could be without ambiguity named as *Leopoldamys neilli*.

## Discussion

### 1. Phylogenetic relationships within the Rattini tribe

#### 1.1. Division-level relationships

Our phylogenetic analyses of Indochinese Rattini based on the combination of cytb, COI and the first exon of the IRBP genes is compatible with the revised taxonomy of Rattini divisions proposed by Musser and Carleton [16]. The *Maxomys *division, the *Dacnomys *division (here consisting of *Leopoldamys *and *Niviventer *as sister taxa) and the *Rattus *division (here including the genera *Rattus*, *Bandicota *and *Berylmys*) are sustained with the highest support values (Figure 2). These results are congruent with the Murinae phylogeny obtained by Lecompte *et al. *[17] based on the analysis of the combined cytb, IRBP and GHR genes. In this latter analysis, the 3 divisions are well supported and the *Maxomys *division is also the first to diverge followed by the *Dacnomys *one and the *Rattus *group sensu stricto of Verneau [64].

#### 1.2. Relationships among the *Rattus* division: is the genus *Rattus* paraphyletic?

In our analyses, the position of *Bandicota *still remains uncertain. The monophyly of the genus *Rattus *is in reality weakly supported (0.82 for pp and 61/63 for Bp) and SH-test failed to reject the hypothesis of a paraphyletic *Rattus *genus (*i.e. Bandicota *is placed within *Rattus*). Verneau and collaborators [64,37] attempted to determine the evolutionary relationships in *Rattus *sensu lato using LINE-1 (L1) amplification events. In their study [37], two LINE subfamilies were identified in the *Bandicota *and the other *Rattus *species except in *Rattus fuscipes*. Since L1 subfamily absence from a particular taxa reflects an ancestral state rather than a derived state [64], these findings excluded *Rattus fuscipes *from a *Bandicota/Rattus *clade and placed *Bandicota *inside the genus *Rattus *leading to its paraphyly. Our study is in agreement with the multi-locus phylogeny of Lecompte *et al.*, [17] which shows *Bandicota *and the genus *Diplothrix *diverging together prior to the *Rattus *clade. In the Lecompte's study, the monophyly of the genus *Rattus *is highly supported (98 Bp, 1 pp) but, as in our study, no specimen of the *Rattus fuscipes *species group was included. To draw conclusions about paraphyly in *Rattus *genus, it would be judicious to complete the taxa sampling among the genus *Rattus *and to include representatives of each *Rattus *species group defined by Musser and Carleton [16] particularly representatives of the *Rattus fuscipes *species group.

#### 1.3. Relationships within the genus *Rattus*

The genus *Rattus*, with a total of 66 species currently recognised [16] "is not only the single largest mammalian genus of all, but also arguably among the most complex and least understood" [65].

Within this genus, *7 *species groups have been defined by Musser and Carleton [16], of which 3 inhabit the Indochinese region and are relevant to this study (Rr, Re and Rn in Figures 2 and 3). The *Rattus rattus *species group as described by Musser and Carleton [16] comprises 21 species of which 5 may be found in Thailand, Cambodia and Lao PDR. In our phylogenetic analysis, this cluster appears unambiguously to be monophyletic (1.00 for pp; 93/97 for Bp) and was placed undoubtedly as the sister group of the monotypic *exulans *species group (pp = 1.00; Bp = 94/96). This association was also found in recent molecular studies [25,17] but encompassing fewer representatives of the *Rattus rattus *species group. According to Musser and Carleton [16], the *R. norvegicus *species group includes 3 species (*Rattus norvegicus*, *R. nitidus *and *R. pyctoris*) of which only 2 may occur in the Indochinese region (*Rattus norvegicus *and *R. nitidus*). This group appears in our study as the sister taxa to the "*R. exulans species *group/*R. rattus *species group" cluster as found in [25] and [17].

Robins and colleagues [25] focusing on rats inhabiting islands in Southeast Asia, included in their sampling specimens from Australia (*i.e. *belonging to the *Rattus fuscipes *species group as defined by [16]) and from New Guinea and adjacent archipelagos (i.e. belonging to the *Rattus leucopus *group). Based on the analysis of nearly 2 kb of *mt *DNA, they recovered 5 of the 7 groups proposed by Musser and Carleton [16]. Our study, even if focusing on a different region of South East Asia, is perfectly congruent with Robins' study, and both studies are compatible with the revised taxonomy of the *Rattus *genus recently proposed by Musser and Carleton [16]. The sixth group defined by the authors [16] corresponds to the *xanthurus *species group encompassing species native to Sulawesi and adjacent islands. According to preliminary phylogenetic analyses of cytb sequences cited in [16], this assemblage could be placed as the sister-group to the *R. leucopus *and *R. fuscipes *groups. The last group defined by Musser and Carleton [16] does not correspond to a natural cluster but was formed for practical reasons since it includes species whose phylogenetic affinities have to be clarified; some may need to be excised from *Rattus*.

### 2. Toward a deep taxonomic revision of the Rattini tribe

At a specific level, we realized that phylogenetic relationships were difficult to discuss. Species misidentifications are indeed plentiful and recurrent both in our sampling (see Table 1) and in the literature. *Mt *sequences from Robins *et al. *[25] or provided by O. Verneau and F. Catzeflis were included in our dataset but questions about the reliability of the identification of vouchers were rapidly raised. To cite a few examples, the *Rattus tanezumi *sample occurring in the *tiomanicus *cluster in [25] (see Figure 4) was proposed by the authors to represent a misidentification. Similarly, the *R. rattus cf. moluccarius *specimen in [64] and [37] was, according to Musser and Carleton [16], an example of *R. nitidus *whereas their specimen assigned to *Niviventer niviventer *was probably improperly identified since *N. niviventer *has never been described in the locality where the specimen was caught [64]. We observed that the situation was worse regarding the *Niviventer *genus. When including sequences available in the databanks (*i.e. *cytochrome *b *sequences from [66]), numerous species appeared to be paraphyletic (data not shown). These results are presumably the consequence of species misidentifications and this explains why we decided to exclude these sequences from our analyses. All in all, these reports ([25,64] and this study) stressed the necessity of a sound taxonomic revision of the Rattini tribe. Consequently one must first determine valid species boundaries and then assign an appropriate name in accordance with the rules of the International Code of the Nomenclature.

#### 2.1. How many rat species in the Indochinese area we investigated?

According to Musser and Carleton [16], 9 genera corresponding to the following 27 species of Rattini may occur in our sampling area (Figure 1): *Hapalomys delacouri *(see *Background *for justification of its inclusion into the Rattini tribe), *Sundamys muelleri*, *Chiromyscus chiropus*, 3 *Maxomys *species (*rajah, surifer, whiteheadi*), 6 *Niviventer *species (*fulvescens, hinpoon, langbianis, tenaster, cremoriventer, confucianus*), 3 *Leopoldamys *species (*neilli, edwardsi, sabanus*), 2 *Bandicota *species (*indica *and *savilei*), 2 *Berylmys *species (*bowersi *and *berdmorei*) and 8 *Rattus *species (*andamanensis, argentiventer, exulans, tanezumi, losea, tiomanicus, norvegicus, nitidus)*. According to our phylogeny (Figure 2), 23 lineages exist within our sampling and 24 putative species were suggested by the method of Pons *et al. *[28]. Confidence interval for the estimated number of species ranged from 22 to 32 (*i.e. *estimates falling within 2 log-likelihood units of the ML solution). An inadequate population sampling is one of the potential limitations of the branch length method as identified by Pons et al. [28]. However, the GMYC model was preferred over the null model of uniform branching rates indicated that the intraspecific sampling effort is satisfactory in our dataset (failure to reject the null model over the GMYC model could be an incomplete sampling per species; [28]). Moreover, among the 24 estimated species, 4 species (labelled R5, Be2b, N2 and N3 respectively in Figure 3) contain a single individual. In accordance with Pons et al, it seems that the GMYC method correctly deals with the inclusion of some rare species represented by only one single individual [28].

The estimated number of species fit well with the number of species described in the literature for this area, although there are some exceptions, in particular within the *Berylmys *and the *Rattus *genera. Our study suggests 3 putative species of *Berylmys *in our sampling whereas only 2 are mentioned in the literature within the geographic area sampled (*Berylmys bowersi *and *B. berdmorei*) (see Table 4). This outcome was supported by all the solutions included in the 95% confidence interval of the estimate of the number of species (Figure 3). This finding may be an artefact of the species delimitation method which could have difficulty in dealing with high level of population differentiation and strong phylogeographic patterns. As acknowledged by Pons *et al.*, [28], a limitation of this method is that populations with partial gene flow risk being recognized as separate entities. A marked phylogeographic structuring within *Berylmys bowersi *could explain the distinction of Be2a and Be2b as two putative species by the branch-length method. Be2b specimen came from the Kanchanaburi locality (Table 1, Figure 1), North to the Isthmus of Kra corresponding to the limit of the peninsular Thailand whereas the specimens of the Be2b group came from the Northern Thailand (Loei and Nan provinces, Figure 1) and Northern Lao PDR (Luang Prabang province, Figure 1). Populations of *Berylmys bowersi *in peninsular Thailand were reported to be geographically isolated and to differ in some ways from other populations [67]. Our findings are congruent with this report. Further investigations are needed to determine if Be2a and Be2b are two phylogenetic lineages of a same species exhibiting a strong phylogeographical pattern or if they have two be considered as two closely related but separate species.

In a similar way, five species belonging to the *Rattus rattus *species group have been described in this area (*i.e. R. andamanensis, argentiventer, tanezumi, losea*, and *tiomanicus*). Marshall [33] reported also the presence of *R. rattus *in all provinces of Thailand and considered the roof rat as the most abundant mammal in the country. Interestingly, since 1998, no specimen among the 3,000 caught during our successive field surveys in rural or urban areas of Thailand, Lao PDR and Cambodia could be identified as a representative of *R. rattus*, according to morphological, cytological and molecular evidences. Our findings offer no support for the presence of *R. rattus *in the area and are in conflict with previous claims of *R. rattus *in the Indochinese region [33]. However, this inconsistancy is probably due to a difference in the usage of "*Rattus rattus*" in place of "*Rattus tanezumi*" rather than a problem of identification or occurrence.

**Figure 5:**
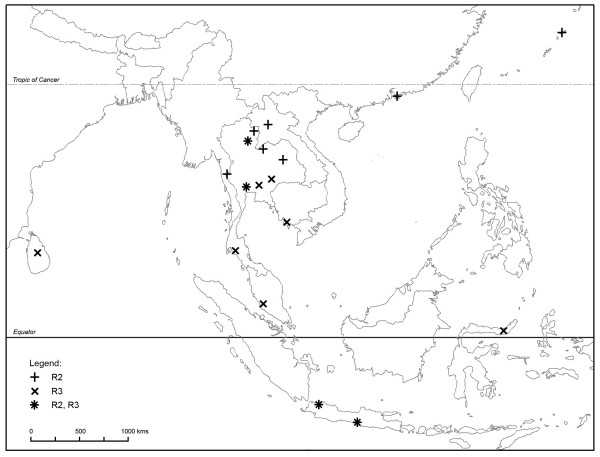
**Map of the distribution of the two Asian species of the *Rattus rattus *species group, according to the samples identified as belonging to R2 and R3 in our study**. (Figures 3 and 4).

Finally, our analysis corroborates the presence of an additional *Rattus *species (labelled R3 in Figure 3) already identified as the *diardii *clade in the mitochondrial phylogeny of Robins et al. [25]. R3 could be a cryptic species. This statement yet needs further investigation using independent data (morphology, nuclear genes). Then, if this hypothesis proved to be correct, the R3 species would have to be carefully named (*R. diardii *is indeed considered at present as a synonym of *R. tanezumi *[16]). In agreement with our result, Aplin in his preliminary study of the cytb [65] observed that the taxonomy of the *Rattus rattus *species group might be rather thornier than suggested by previous studies mostly based on karyotypic or electrophoretic evidences. Indeed, his ongoing study reports two distinct phylogenetic clades in the Asian region. The first one would correspond to an endemic Southeast Asian taxon (recorded in Vietnam, Cambodia and Southern Laos) and might correspond to our R3 according to geographical evidence. Our study and Robins' work reveal that the distribution of this Southeast group spreads far into the South as it occurs in Thailand and in Sri Lanka and also in Malaysia, in Indonesia and Northern Sulawesi (Figures 4 and 5). The second clade proposed by Aplin [65] would be a northern and South Asian taxon (found in Japan, Hong Kong, northern Vietnam, northern Laos, and Bangladesh) and might correspond to R2 (here also found in Thailand and Indonesia, Figure 4, Table 3/see also Table 4 for species name). Indeed when including Robins' sequences, R2 includes specimens from Japan and Hong Kong (Figures 4 and 5). As mentioned by Aplin [65], the latter group (R2) is more closely related to *Rattus rattus *rather than the former group (R3). In our trees (Figures 2 and 4), R2 is clearly placed as the sister taxa of *R. rattus *(R1). Our study reinforces Aplin's assumption [65] that the two Asian clades (*i.e. *R2 and R3) are sympatric in some part of their distribution by increasing greatly the area where the two taxa co-occur in continental Southeast Asia. Both are found in Northern and Central Thailand (Phrae, Nakhon Pathom and Ratchaburi provinces; this study). Since some specimens of both taxa were trapped in exactly the same location and time, at least in Phrae, they probably also share similar habitats and are likely syntopic.

#### 2.2. How to give a name?

By integrating phylogenetic, morphological and geographical evidence, we proposed to attribute the names summarized in Table 4 to the 24 species highlighted herein. Our propositions are not definitive but are revisable ones. Indeed, once species boundaries are delimitated, assigning the appropriate name to each species is not an easy task particularly for the Rattini species whose taxonomy is complicated by a large number of synonym names. Even for a rodent specialist, morphological characters are sometimes misleading (see aforementioned misidentification examples) and intraspecific morphological polymorphism makes the problem more difficult. To alleviate this last difficulty, morphological studies have to consider a large number of specimens, a process that may be difficult and time-consuming to perform.

These inconveniences highlighted the great interest in obtaining molecular data from a holotype. Indeed, the holotype is by definition the element to which the name of a taxon is permanently attached. Consequently, including holotype specimens in molecular phylogenies would be very suitable to name each cluster recognized as a valid species providing that a rigorous and sound taxonomy is already set up. Indeed, holotype specimens may correspond to problematic taxa (*e.g*. problems of synonymy not yet revealed), and the use of type specimens could be misleading in such context. Including holotype specimens in molecular phylogenies is however totally infeasible for the two following reasons. Firstly, holotype specimens are unique and are difficult to obtain for genetic research purposes. Sampling authorisations are very scarce and destructive sampling is generally not possible. To achieve our study, no more than 24 holotypes would be damaged if our assumptions are correct. Faced with the understandable reluctance of museum curators, non-destructive extraction procedure [68] would be an elegant suggestion. Secondly, ancient materials contain tiny amounts of poorly preserved and highly fragmented DNA. As required for this study, getting 3 kb corresponding to 3 different genes (including one nuclear one) for more than 24 holotype specimens, and following the ancient DNA guidelines would be too expensive and much too time-consuming. To circumvent this problem it is fortunately possible to target small DNA fragments as barcodes. Our study proved that this strategy is a powerful one. Following all the ancient DNA requirements, we succeeded in amplifying a genuine small cytb fragment from the *Leopoldamys neilli *holotype. This barcode was used to assign a name without ambiguity to one of the clusters (*i.e. *L2) recognized as a valid species in our analyses. Even if more holotype specimens have to be investigated to achieve a steady revision of the Rattini tribe, our work illustrates the huge opportunities ancient DNA analysis may offer to taxonomists.

## Conclusions

This study represents the first step of a long-term project aiming at a deep taxonomic revision of the Rattini. Putative species delimitations have been determined here without prior assumptions and we propose a suitable methodology using molecular data from holotypes to assign the right name to each delineated species. Ancient DNA analysis of holotypes should be considered by taxonomists as a promising tool opening up new realms of possibilities (*e.g. *testing synonymy of names of unclear taxonomies such as the synonymy of *R. tanezumi *and *R. diardii*; see Table 4). Although DNA data alone are not a panacea for species description and delimitation, we are confident that future investigations combined with other types of information will clarify the taxonomy of this confusing group. Indeed, integrative approaches merging independent data such as morphology, karyology, mitochondrial and nuclear markers are the only means to understand the diversification among, and interactions between, evolutionary lineages. Our molecular study revealed that at least 7 putative different species, including a cryptic one (R3), could exist among the *Rattus rattus *species group (among which six were sampled within the area we investigated). As each of these species is expected to have specific ecological traits and to carry its own set of diseases, the recognition of cryptic species within Rattini could have serious implications for human health in Southeast Asia. However, this result has to be carefully considered. Indeed, it is worth noticing that the terminal nodes of our multilocus phylogeny are mostly supported by mitochondrial data (cytb and COI genes) while the deepest nodes are sustained by nuclear data (IRPB). Other kinds of markers have thus to be checked for congruence. Such clarifications for the Rattini tribe are today urgently required to achieve meaningful epidemiological research in South East Asia.

## List of Abbreviations

bp: base pairs; kb: kilo base pairs.

## Authors' contributions

Conceived and designed the experiments: JM, JFC, MP. Performed the experiments: MP, YC, VH, SW. Analyzed the data: MP. Wrote the paper: MP, YC. Senior epidemiologists and supervisors responsible for all scientific output of the program: SM, JM, JPH. All authors read and approved the final manuscript.

## Supplementary Material

Additional file 1 **Rat 85 pb cytb alignment**. The whole cytb sequences obtained from the 122 specimens selected in this study were reduced to the 85 bp DNA marker already used to discriminate closely related species from degraded DNA [56,57]. Small sequences were sorted following the results of the DNA-based species delimitation method. Dots indicate identical positions as those of the reference sequence of *Rattus rattus *R12. Sites allowing discrimination between species are those shared by all the specimens of a same entitie but different for all the specimens of another one. Each rat species could be distinguished from each other based on this fragment except the two *Berylmys *species Be2a and Be2b (but see discussion). We tried to maximize the geographic diversity of the specimens, however, our sampling was achieved without prior expectation and some entities determined by the DNA-based species delimitation method encompass few specimens coming from the same locality (e.g. R5, R6, R7, Be2b, L3, etc.). In this case, intra-polymorphism is not taken into account and substitutions allowing discriminatation between species are thus overestimated. However, closely related rat species (such as R1 and R2 or R3 and R4, see phylogeny in Figure 2) could be easily discriminated. We thus considered that this fragment is reliable for an adequate discrimination for rat species. Click here for file

Additional file 2 **Lineage-through-time plot based on the Multidivtime ultrametric tree**. The sudden increase in branching rate, indicated by a red line, corresponds to the shift from interspecific to intraspecific lineage branching. Click here for file

## References

[B1] Morand S Krasnov BR Poulin R Micromammals and macroparasites: from evolutionary ecology to management 2006 Tokyo: Springer-Verlag

[B2] Herbreteau V Henttonen H Yoshimatsu K Gonzalez JP Suputtamongkol Hugot JP Tibayrenc, M Hantavirus coevolution with their rodent hosts Encyclopedia of infectious diseases. Modern methodologies 2007 1 Wiley J & Sons Inc 243 264 full_text

[B3] Henttonen H Buchy P Suputtamongkol Y Jittapalapong S Herbreteau V LaakkonenJ Chaval Y Galan M Dobigny G Charbonnel N Michaux J Cosson JF Morand S Hugot JP Recent discoveries of new hantaviruses widen their range and question their origins Ann N Y Acad Sci 2008 1149 84 89 10.1196/annals.1428.064 19120180

[B4] Jittapalapong S Herbreteau V Hugot JP Arreesrisom P Karnchanabanthoeng A Rerkamnuaychoke W Morand S Relationship of parasites and pathogens diversity to rodents in Thailand Kasetsart J 2009 43 106 117

[B5] Meerburg B Singleton G Kijlstra A Rodent-borne diseases and their risks for public health Critical Reviews in Microbiology 2009 35 221 270 10.1080/10408410902989837 19548807

[B6] Ostfeld R Keesing F Biodiversity and disease risk: the case of Lyme disease Conserv Biol 2000 14 722 728 10.1046/j.1523-1739.2000.99014.x

[B7] Suzán G Marcé E Giermakowski JT Mills JN Ceballos G Ostfeld RS Armien B Pascale JM Yates TL Experimental evidence for reduced rodent diversity causing increased hantavirus prevalence PLoS ONE 2009 4 e5461 10.1371/journal.pone.0005461 19421313 PMC2673579

[B8] Chivian E Biodiversity: its importance to human health 2003 Boston, Massuschets Harvard Medical School

[B9] Morand S Krasnov B Poulin R Morand S, Krasnov BR, Poulin R Global change, biodiversity and the future of mammals-parasite interactions Micro-mammals and macroparasites: from evolutionary ecology to management 2006 Tokyo: Springer-Verlag 617 635

[B10] de la Rocque S Morand S Hendrix G Climate change and pathogens Rev Sci Tech Office International des Epizooties 2008 27

[B11] Wilson D Reeder D Johns Baltimore Mammal Species of the World. A Taxonomic and Geographic Reference 2005 3 Maryland Hopkins University Press

[B12] Jenkins PD Kilpatrick W Robinson M Timmins R Morphological and molecular investigations of a new family, genus and species of rodent (Mammalia: Rodentia: Hystricognatha) from Lao PDR System Biodivers 2005 2 419 454 10.1017/S1477200004001549

[B13] Musser G Smith A Robinson MF Lunde D Description of a new genus and species of rodent (Murinae, Muridae, Rodentia) from the Khammouan limestone national biodiversity conservation area in Lao PDR Am Mus Novit 2005 3497 1 31 10.1206/0003-0082(2005)497[0001:DOANGA]2.0.CO;2

[B14] Helgen KM A new species of murid rodent (genus *Mayermys*) from South-eastern New Guinea Mamm Biol 2005 70 61 67 10.1078/1616-5047-00176

[B15] Musser G Lunde D Truong Son N Description of a new genus and species of rodent (Murinae, Muridae, Rodentia) from the lower karst region of Northeastern Vietnam Am Mus Novit 2006 3571 1 41 10.1206/0003-0082(2006)3517[1:DOANGA]2.0.CO;2

[B16] Musser G Carleton M Wilson DE, Reeder DM Superfamily Muroidea Mammal species of the world: A taxonomic and geographic reference 2005 2 3 Baltimore: Johns Hopkins University 894 1531

[B17] Lecompte E Aplin K Denys C Catzeflis F Chades M Chevret P Phylogeny and biogeography of African Murinae based on mitochondrial and nuclear gene sequences with a new tribal classification of the subfamily BMC Evol Biol 2008 8 199 10.1186/1471-2148-8-199 18616808 PMC2490707

[B18] Myers N Mittermeier RA Mittermeier CG da Fonseca GA Kent J Biodiversity hotspots for conservation priorities. Nature 2000 403 853 858 10.1038/35002501 10706275

[B19] Matsui S Protecting human and ecological health under viral threats in Asia Water Sci Technol 2005 51 91 97 16007933

[B20] Forman S Hungerford N Yamakawa M Yanase T Tsai HJ Joo YS Yang DK Nha JJ de la Rocque S, Morand S, Hendrix G Climate change impacts and risks for animal health in Asia Climate change and pathogens Rev Sci Tech, Office International des Epizooties 27 2008 581 597 18819679

[B21] Rowe KC Reno ML Richmond DM Adkins RM Steppan SJ Pliocene colonization and adaptive radiations in Australia and New Guinea Sahul Multilocus systematics of the old endemic rodents Muroidea Murinae Mol Phylogenet Evol 2008 47 84 101 10.1016/j.ympev.2008.01.001 18313945

[B22] Xiao S LeDuc J Chu Y Schmaljohn C Phylogenetic analyses of virus isolates in the genus Hantavirus, family Bunyaviridae Virology 1994 198 205 217 10.1006/viro.1994.1023 8259656

[B23] Hugot JP Plyusnina A Herbreteau V Nemirov K Laakkonen Lundkvist A Supputamongkol Y Henttonen H Plyusnin A Genetic analysis of Thailand hantavirus in *Bandicota indica *trapped in Thailand Virol J 2006 3 72 81 10.1186/1743-422X-3-72 16953877 PMC1578555

[B24] Coleman RE Monkanna T Linthicum KJ Strickman DA Frances SP Tanskul P Kollars TM Jr Inlao I Watcharapichat P Khlaimanee N Phulsuksombati D Sangjun N Lerdthusnee K Occurrence of *Orientia tsutsugamushi *in small mammals from Thailand Am J Trop Med Hyg 2003 69 519 524 14695089

[B25] Robins J Hingston M Matisoo-Smith E Ross H Identifying *Rattus *species using mitochondrial DNA Mol Ecol Notes 2007 7 717 729 10.1111/j.1471-8286.2007.01752.x

[B26] Badenhorst D Herbreteau V Chaval Y Pagès M Robinson TJ Rerkamnuaychoke W Morand S Hugot JP Dobigny G New karyotypic data for Asian rodents (Rodentia, Muridae) with the first report of B-chromosomes in the genus *Mus* J Zool 2009 doi:10.1111/j.1469-7998.2009.00588.x

[B27] Bradley R Baker R A test of the genetic species concepts: cytochrome*-b *sequences and mammals J Mammal 2001 82 960 973 10.1644/1545-1542(2001)082<0960:ATOTGS>2.0.CO;2

[B28] Pons J Barraclough TG Gomez-Zurita J Cardoso A Duran DP Hazell S Kamoun S Sumlin WD Vogler AP Sequence-based species delimitation for the DNA taxonomy of undescribed insects Syst Biol 2006 55 595 609 10.1080/10635150600852011 16967577

[B29] Jousselin E Desdevises Y Coeur d'acier A Fine-scale cospeciation between *Brachycaudus *and *Buchnera **aphidicola*: bacterial genome helps define species and evolutionary relationships in aphids Proc Biol Sci 2009 276 187 196 10.1098/rspb.2008.0679 18782748 PMC2614242

[B30] Fontaneto D Herniou E Boschetti C Caprioli M Melone G Ricci C Barraclough TG Independently evolving species in asexual bdelloid rotifers PLoS Biol 2007 5 e87 10.1371/journal.pbio.0050087 17373857 PMC1828144

[B31] Fontaneto D Boschetti C Ricci C Cryptic diversification in ancient sexual: evidence from the bdelloid rotifer *Philodina flaviceps* J Evol Biol 2008 21 580 587 10.1111/j.1420-9101.2007.01472.x 18081746

[B32] Bickford D Lohman D Sodhi N Ng P MeierR Cryptic species as a window on diversity and conservation Trends Ecol Evol 2006 22 148 155 10.1016/j.tree.2006.11.004 17129636

[B33] Marshall JD Lekagul B, McNeely JA Rats and mice of Thailand Mammals of Thailand 1977 Saha Karn Bhaet Bangkok, Thailand 395 490

[B34] Corbet G Hill J The Mammals of the Indomalayan Region: A Systematic Review 1992 Oxford University Press, USA

[B35] Aplin KP Brown PR Jacob J Krebs CJ Singleton GR Field methods for rodent studies in Asia and the Indo-Pacific 2003 Canberra: Australian Centre for International Agricultural Research

[B36] Tollenaere C Brouat C Duplantier JM Rahalison L Rahelinirina S Pascal M Moné H Mouahid G Leirs H Cosson JF Phylogeography of the invasive species *Rattus rattus *in the western Indian Ocean, with special emphasis on the colonization history of Madagascar J Biogeogr 2010 37 398 410 10.1111/j.1365-2699.2009.02228.x

[B37] Verneau O Catzeflis F Furano AV Determining and dating recent rodent speciation events by using L1, LINE-1 retrotransposons Proc Natl Acad Sci USA 1998 95 11284 11289 10.1073/pnas.95.19.11284 9736728 PMC21634

[B38] Michaux J Chevret P Renaud S Morphological diversity of Old World rats and mice (Rodentia, Muridae) mandible in relation with phylogeny and adaptation J Zoolog Syst Evol Res 2007 45 263 279 10.1111/j.1439-0469.2006.00390.x

[B39] Jansa SA Weksler M Phylogeny of muroid rodents: relationships within and among major lineages as determined by IRBP gene sequences Mol Phylogenet Evol 2004 31 256 276 10.1016/j.ympev.2003.07.002 15019624

[B40] Jansa SA Barker FK Heaney LR The pattern and timing of diversification of Philippine endemic rodents: evidence from mitochondrial and nuclear gene sequences Syst Biol 2006 55 73 88 10.1080/10635150500431254 16507525

[B41] Galtier N Gouy M Gautier C SEAVIEW and PHYLO_WIN: two graphic tools for sequence alignment and molecular phylogeny Comput Appl Biosci 1996 12 543 548 9021275 10.1093/bioinformatics/12.6.543

[B42] Rice P Longden I Bleasby A EMBOSS: The European Molecular Biology Open Software Suite Trends Genet 2000 16 276 277 10.1016/S0168-9525(00)02024-2 10827456

[B43] Philippe H Delsuc F Brinkmann H Lartillot N Phylogenomics Annual Review of Ecology, Evolution, and Systematics 2005 36 541 562 10.1146/annurev.ecolsys.35.112202.130205

[B44] Xia X Xie Z DAMBE: software package for data analysis in molecular biology and evolution J Hered 2001 92 371 373 10.1093/jhered/92.4.371 11535656

[B45] Swofford DL PAUP*. Phylogenetic Analysis using Parsimony, * and other methods 1998 Sunderland, Massachusetts Sinauer Associates Version 4

[B46] Phillips M Delsuc F Penny D Genome-scale phylogeny and the detection of systematic biases Mol Biol Evol 2004 21 1455 1458 10.1093/molbev/msh137 15084674

[B47] Nylander JAA MrAIC.pl 2004 Program distributed by the author. Evolutionary Biology Centre, Uppsala University

[B48] Guindon S Gascuel O A simple, fast, and accurate algorithm to estimate large phylogenies by maximum likelihood Syst Biol 2003 52 696 704 10.1080/10635150390235520 14530136

[B49] Stamatakis A RAxML-VI-HPC: maximum likelihood-based phylogenetic analyses with thousands of taxa and mixed models Bioinformatics 2006 22 2688 2690 10.1093/bioinformatics/btl446 16928733

[B50] Stamatakis A Hoover P Rougemont J A rapid bootstrap algorithm for the RAxML Web servers Syst Biol 2008 57 758 771 10.1080/10635150802429642 18853362

[B51] Ronquist F Huelsenbeck JP MrBayes 3: Bayesian phylogenetic inference under mixed models Bioinformatics 2003 19 1572 1574 10.1093/bioinformatics/btg180 12912839

[B52] Rambaut A Drummond A Tracer v1.4 2003 http://beast.bio.ed.ac.uk/Tracer

[B53] Shimodaira H Hasegawa M Multiple comparisons of log-likelihoods with applications to phylogenetic inference Mol Biol Evol 1999 16 1114 1116

[B54] Thorne JL Kishino H Divergence time and evolutionary rate estimation with multilocus data Syst Biol 2002 51 689 702 10.1080/10635150290102456 12396584

[B55] Rutschmann F Bayesian molecular dating using PAML/MULTIDIVTIME. A step-by-step manual 2005 http://statgen.ncsu.edu/thorne/multidivtime.html Version 1.415878126

[B56] Télétchéa F Bernillon J Duffraisse M Laudet V Hänni C Molecular identification of vertebrate species by oligonucleotide microarray in food and forensic samples J Appl Ecol 2008 45 967 975 10.1111/j.1365-2664.2007.01415.x

[B57] Pagès M Desse-Berset N Brosse L Hänni C Berrebi P Historical presence of the sturgeon *Acipenser sturio *in the Rhône basin determined by the analysis of ancient DNA cytochrome *b *sequences Conserv Genet 2009 10 217 224 10.1007/s10592-008-9549-6

[B58] Hughes S Hayden TJ Douady CJ Tougard C Germonpré M Stuart A Lbova L Carden RF Hänni C Say L Molecular phylogeny of the extinct giant deer, Megaloceros giganteus Mol Phylogenet Evo 2006 40 285 291 10.1016/j.ympev.2006.02.004 16556506

[B59] Gilbert MT Bandelt HJ Hofreiter M Barnes I Assessing ancient DNA studies Trends Ecol Evol 2005 20 541 544 10.1016/j.tree.2005.07.005 16701432

[B60] Rohland N Hofreiter M Ancient DNA extraction from bones and teeth Nat Protoc 2007 2 1756 1762 10.1038/nprot.2007.247 17641642

[B61] Sarkar I Planet P DeSalle R CAOS Software for use in character based DNA barcoding Mol Ecol Resour 2008 8 1256 1259 10.1111/j.1755-0998.2008.02235.x 21586014

[B62] Hofreiter M Jaenicke V Serre D Haeseler Av A Pääbo S DNA sequences from multiple amplifications reveal artifacts induced by cytosine deamination in ancient DNA Nucleic Acids Res 2001 29 4793 4799 10.1093/nar/29.23.4793 11726688 PMC96698

[B63] Gilbert MT Hansen AJ Willerslev E Rudbeck L Barnes I Lynnerup N Cooper A Characterization of genetic miscoding lesions caused by postmortem damage Am J Hum Genet 2003 72 48 61 10.1086/345379 12489042 PMC420012

[B64] Verneau O Catzeflis F Furano AV Determination of the evolutionary relationships in *Rattus sensu lato *(Rodentia: Muridae). using L1, LINE-1. amplification events J Mol Evol 1997 45 424 436 10.1007/PL00006247 9321421

[B65] Aplin KP Chesser T ten Have J Singleton GR, Hinds LA, Krebs CJ, Spratt DM Evolutionary biology of the genus *Rattus*: a profile of an archetypal rodent pest Rats, mice and people: rodent biology and management 2003 Canberra: Australian Centre for International Agricultural Research 487 498

[B66] Jing M Yu HT Wu SH Wang W Zheng X Phylogenetic relationships in genus *Niviventer*, Rodentia: Muridae in China inferred from complete mitochondrial cytochrome *b *gene Mol Phylogenet Evol 2007 44 521 529 10.1016/j.ympev.2007.04.003 17531508

[B67] Francis CM A field guide to the mammals of South-East Asia 2008 London: New Holland

[B68] Asher RJ Hofreiter M Tenrec phylogeny and the noninvasive extraction of nuclear DNA Syst Biol 2006 55 181 194 10.1080/10635150500433649 16522569

[B69] Irwin DM Kocher TD Wilson AC Evolution of the cytochrome *b *gene of mammals J Mol Evol 1991 32 128 144 10.1007/BF02515385 1901092

[B70] Poux C Douzery EJ Primate phylogeny, evolutionary rate variations, and divergence times: a contribution from the nuclear gene IRBP Am J Phys Anthropol 2004 124 1 16 10.1002/ajpa.10322 15085543

[B71] Ellerman JR The Families and Genera of Living Rodents 1941 London, The British Museum Natural History

[B72] Jentink F On a new genus and species of *Mus *from Madagascar Notes of the Leyden Museum 1879 107 109

[B73] Catzeflis F Animal tissue collections for molecular genetics and systematics Trends Ecol Evol 1991 6 168 10.1016/0169-5347(91)90060-B 21232449

[B74] Aplin KP Frost A Tuan NP Lan LP Hung NM Singleton GR, Hinds LA, Krebs C J, Spratt DM Identification of rodents of the genus *Bandicota *in Vietnam and Cambodia Rats, mice and people: rodent biology and management 2003 Canberra: Australian Centre for International Agricultural Research 531 535

[B75] Musser G Newcomb C Malaysian murids and the giant rat of Sumatra Bull Am Mu Nat Hist 1983 174 327 598

[B76] Lunde D Aplin K Leopoldamys neilli IUCN 2008 http://www.iucnredlist.org IUCN Red List of Threatened Species. Downloaded on 13 March 2009

[B77] Musser G Results of the Archbold expeditions N°105. Notes on systematics of Indo-malayan murid rodents, and descriptions of new genera and species from Ceylon, Sulawesi, and Philippines Bull Am Mus Nat Hist 1981 168 225 334

[B78] Gorog A Sinaga M Engstrom M Vicariance or dispersal? Historical biogeography of three Sunda shelf murine rodents (*Maxomys surifer Leopoldamys sabanus and Maxomys whiteheadi*) Biol J Linn Soc Lond 2004 81 91 109 10.1111/j.1095-8312.2004.00281.x

