# Supplementary material for: Revisiting the taxonomy of the Rattini tribe: a phylogeny-based delimitation of species boundaries
Source: BMC Evol Biol. 2010 Jun 18;10:184. doi: 10.1186/1471-2148-10-184 (PMC2906473; doi:10.1186/1471-2148-10-184)

**Additional File, S1: Rat 85pb cytb alignment**

The whole cytb sequences obtained from the 122 specimens selected in this study were reduced to the 85bp DNA marker already used to discriminate closely related species from degraded DNA [56-57]. Small sequences were sorted following the results of the DNA-based species delimitation method. Dots indicate identical positions as those of the reference sequence of *Rattus rattus* R12. Sites allowing discrimination between species are those shared by all the specimens of a same entity but different for all the specimens of another one. Each rat species could be distinguished from each other based on this fragment except the two *Berylmys* species Be2a and Be2b (but see discussion).

We tried to maximize the geographic diversity of the specimens, however, our sampling was achieved without prior expectation and some entities determined by the DNA-based species delimitation method encompass few specimens coming from the same locality (e.g. R5, R6, R7, Be2b, L3, etc.). In this case, intra-polymorphism is not taken into account and substitutions allowing discrimination between species are thus overestimated. However, closely related rat species (such as R1 and R2 or R3 and R4, see phylogeny in Figure 2) could be easily discriminated. We thus considered that this fragment is reliable for an adequate discrimination for rat species.


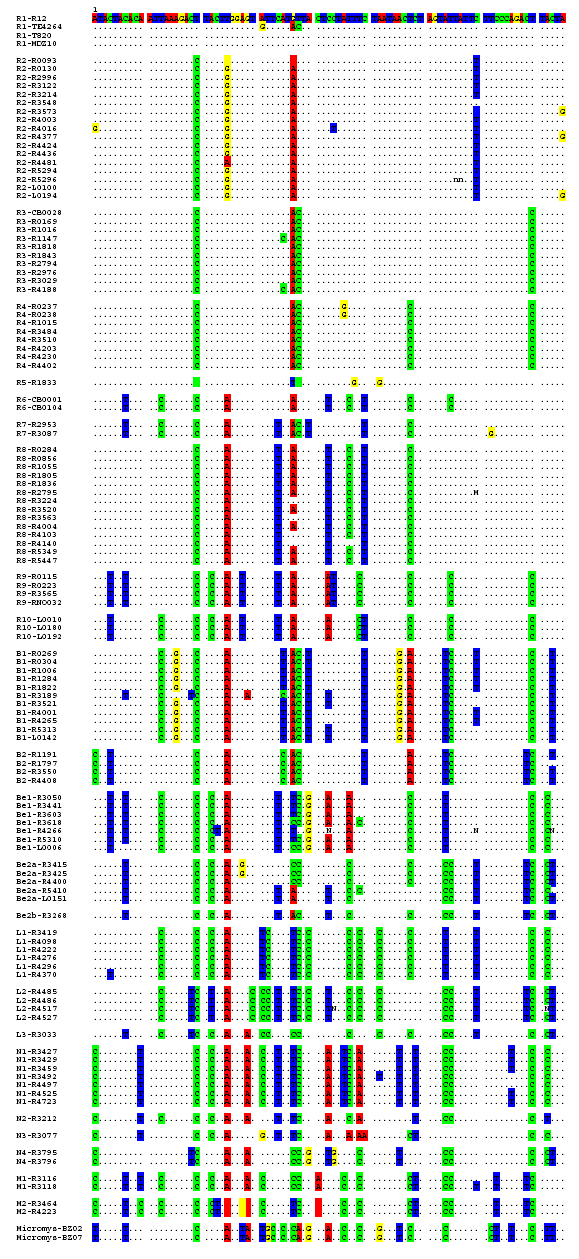

Supplement: Additional file 1 — Rat 85 pb cytb alignment. The whole cytb sequences obtained from the 122 specimens selected in this study were reduced to the 85 bp DNA marker already used to discriminate closely related species from degraded DNA [56,57]. Small sequences were sorted following the results of the DNA-based species delimitation method. Dots indicate identical positions as those of the reference sequence of Rattus rattus R12. Sites allowing discrimination between species are those shared by all the specimens of a same entitie but different for all the specimens of another one. Each rat species could be distinguished from each other based on this fragment except the two Berylmys species Be2a and Be2b (but see discussion). We tried to maximize the geographic diversity of the specimens, however, our sampling was achieved without prior expectation and some entities determined by the DNA-based species delimitation method encompass few specimens coming from the same locality (e.g. R5, R6, R7, Be2b, L3, etc.). In this case, intra-polymorphism is not taken into account and substitutions allowing discriminatation between species are thus overestimated. However, closely related rat species (such as R1 and R2 or R3 and R4, see phylogeny in Figure 2) could be easily discriminated. We thus considered that this fragment is reliable for an adequate discrimination for rat species. [file 1471-2148-10-184-S1.DOC]
