# Supplementary material for: Revisiting the taxonomy of the Rattini tribe: a phylogeny-based delimitation of species boundaries
Source: BMC Evol Biol. 2010 Jun 18;10:184. doi: 10.1186/1471-2148-10-184 (PMC2906473; doi:10.1186/1471-2148-10-184)
Supplement: Additional file 2 — Lineage-through-time plot based on the Multidivtime ultrametric tree. The sudden increase in branching rate, indicated by a red line, corresponds to the shift from interspecific to intraspecific lineage branching. [file 1471-2148-10-184-S2.DOC]

**Additional file, S2**: Lineage-through-time plot based on the Multidivtime ultrametric tree.

The sudden increase in branching rate, indicated by a red line, corresponds to the shift from interspecific to intraspecific lineage branching.

-0.5

-0.4

-0.3

-0.2

-0.1

0.0

Time

Time (length from root)

-0.4

-0.2

-0.1

100

50

20

10

5

2

1

Number of Lineages

-0.5

-0.3

0.0
